# Developing a culturally tailored short message service (SMS) intervention for improving the uptake of cervical cancer screening among Ghanaian women in urban communities

**DOI:** 10.1186/s12905-022-01719-9

**Published:** 2022-05-10

**Authors:** Harriet Affran Bonful, Adolphina Addoley Addo-Lartey, Ransford Selasi Sefenu, Adanna Nwameme, Timothy Agandah Abagre, Adolf Kofi Awua, Nii Armah Adu-Aryee, Florence Dedey, Richard Mawuena Kofi Adanu, Kolawole Stephen Okuyemi

**Affiliations:** 1 Department of Epidemiology and Disease Control, School of Public Health, University of Ghana, Accra, Ghana; 2 Department of Social and Behavioral Sciences, School of Public Health, University of Ghana, Accra, Ghana; 3 Cellular and Clinical Research Centre, Radiological and Medical Sciences Research Institute, Ghana Atomic Energy Commission, Kwabenya, Accra, Ghana; 4 Department of Surgery, School of Medicine and Dentistry, University of Ghana, Accra, Ghana; 5 Department of Population and Family Health, School of Public Health, University of Ghana, Accra, Ghana; 6 Department of Family and Preventive Medicine, University of Utah, Salt Lake City, USA

**Keywords:** Cervical cancer, Screening, Pap smear, Visual inspection with acetic acid (VIA), Barriers, Facilitators, SMS text message, Ghana

## Abstract

**Background:**

There has been extensive research across the globe to understand the barriers and facilitators of cervical cancer (CC) screening. However, few studies have focused on how such information has been used to develop text messages for mHealth screening programs, especially in resource-poor countries. This study elicited information on barriers and facilitators, the preferences of women regarding the modalities for delivery of health SMS messages on screening for cervical cancer, and demonstrates how this information was used to create a health screening program among women in the Greater Accra Region of Ghana.

**Methods:**

Four main activities were carried out, including (1) a total of five focus group discussions, (2) a baseline survey involving 62 female bankers and 68 women from the communities, (3) a stakeholder meeting involving experts in cervical cancer research and clinical care, and (4) pilot testing of the text messages. Focus group discussions and the baseline survey data were collected concurrently between February and May 2017 and the results were used to develop 5 specific communication objectives during the stakeholder engagements held in June 2017.

**Results:**

In all, 32 text messages were developed and pretested in July 2017(13 addressed knowledge on CC; 6 highlighted the importance of early detection; 5 allayed fear as a barrier to CC screening; 5 encouraged women to have time for their health, and 3 messages contained information on where to go for screening and the cost involved). Although awareness about the disease was high, knowledge of CC screening was low. For two-thirds of respondents (22/33), perceived lack of time, high cost, and fear (of cc, screening procedure, and potential for negative outcome) accounted for the reasons why respondents will not go for screening, while education on CC, especially from health workers and the mass media enabled uptake of CC screening.

**Conclusion:**

Several factors prevent women from accessing screening services for CC, however, barriers such as low levels of education on CC, lack of time, and fear can be targeted in SMS messaging programs.

**Supplementary Information:**

The online version contains supplementary material available at 10.1186/s12905-022-01719-9.

## Introduction

Each year, about half a million women globally develop cervical cancer, and about 274,000 die from the disease, with approximately 80% of cervical cancer deaths occurring in developing countries [1–3]. In Ghana, cervical cancer is the leading cause of cancer-related deaths in women and is most frequent among women aged 15–44 years, with an estimated crude incidence rate of 26.4/100,000/year [4]. Ghana has over 8 million women aged 15 years and older who are at risk of developing cervical cancer [5]. Current estimates indicate that annually more than 3,000 Ghanaian women are diagnosed with cervical cancer, and over 2,000 women die from the disease [4]. There is a critical need to establish screening for cervical cancer, which has been successfully employed in most developed countries to control the disease for over 50 years [6–8]. Screening of women between the ages of 35 and 40 once or twice lowers the lifetime risk of cervical cancer by 25%-35%, while up to three screenings reduce it by 50% [9].

Ghana’s National Reproductive Health Policy implemented a national policy on cervical cancer prevention in 2005 that recommended visual inspection with acetic acid (VIA) screening with the treatment of pre-cancerous lesions with cryotherapy for women aged 25–45 and Papanicolaou (Pap) screening (cytology) for women age 45 and above [4, 10]. Reproductive health nurses were successfully trained in VIA and found to effectively screen patients in a single visit [11]. However, the National Screening Program has stalled, and only a few urban health centers, including Korle Bu Teaching Hospital (KBTH), Ridge Hospital, and Kumasi South Hospital, are currently actively using VIA to screen for cervical cancer [4, 12]. Despite this successful pilot program and the continued availability of viable cervical cancer screening modalities, the screening rate remains unacceptably low in Ghana [4]. The second wave of the World Health Organization (WHO) *Study on global AGEing and adult health *(*SAGE*) found that on average, only 2 in every 100 women (2.4%) in Ghana had ever received a Pap test [13], only a marginal increase in the screening rate (2.1%) reported over a decade ago [14]. This slow adoption of cervical cancer screening reflects common barriers encountered in the global fight against cervical cancer, chief among which are hesitations among women to go for screening. Reasons for this hesitancy are varied but can be chiefly categorized into one of three general reasons. The first of these is fear, whether of pain [15] associated with testing, embarrassment or shame, or discovering a serious disease. Another barrier is a lack of interest in screening, which typically stems from the absence of symptoms [16] or low perceived risk for the disease. Finally, external factors such as neglect, lack of financial resources, lack of time, and lack of encouragement from partners [17–20] have been observed to lower cervical cancer screening rates. In Ghana, for example, cervical cancer screening and treatment services are not covered under the benefits packages of the Ghanaian National Health Insurance Scheme, thus women pay out of pocket before accessing these services.

To overcome these challenges, several approaches to encourage women to embrace screening services have been globally tested, including radio campaigns [17] to raise awareness of screening services as well as reminders to undergo screening in media such as posts, emails, telephone calls, and text messages [21–23]. While these reminder strategies have seen varying success rates in different settings, the most effective form in increasing the uptake of Pap smears has been the use of invitation letters (RR = 1.44, 95% CI 1.24–1.52) [24]. However, most resource-constrained settings, including Ghana, experience logistical challenges (e.g. limitations in transport, infrastructure, and personnel) that make it difficult to operationalize letters or emails from health care providers. As a result of these limitations, strategies in Ghana currently focus on opportunistic screening at a physician’s request as well as awareness campaigns sponsored by professional associations, corporate institutions, and philanthropists. However, this approach has failed to yield an adequate response.

We propose to leverage Ghana’s ever-expanding telecommunication services, which offer considerable affordability and accessibility via text messages, to develop and introduce culturally tailored, evidence-based mobile health (mHealth) based interventions [22, 25] to promote Cervical cancer screening uptake.

Previous research has demonstrated that women are willing to accept SMS text message reminders for screening appointments [26]. While some frameworks have been recommended to guide the development of SMS text messaging programs [27], their practical demonstration has yet to be extensively reported. Even significant mHealth effectiveness trials have not reported the processes that led to the development of the text messages. A few studies have described the procedures employed in the creation of text message libraries, but these rely primarily on qualitative data approaches and exclude quantitative verification of hypotheses generated [22, 28, 29]. Our research responds to this knowledge gap by using both qualitative and quantitative approaches to develop culturally appropriate SMS text messages tailored to address barriers, enhance facilitators, and encourage cervical cancer screening uptake in Ghana.

## Methods

### Study design and participants

This article is a methodological compilation of how mobile text messages were developed and pre-tested for use in a quasi-experimental trial to assess the effectiveness of a culturally tailored program to promote cervical cancer screening in Accra, Ghana. This paper focuses on the processes that led to the development of SMS text messages. The main outcome of the study was the development of SMS text messages for promoting cervical cancer screening. The study utilized a mixed-methods design. A needs assessment was conducted via focus groups paired with a survey to obtain demographics, a basic health history, and knowledge and perceptions regarding cervical cancer screening. Following analysis of these data, the messages were developed in consultation with stakeholder engagements and pre-tested with a small randomly selected group of women.

The sample size for the survey was estimated based on the following assumptions:

1. All women included in the study have never had a pap smear and/or mammogram done
2. Uptake of opportunistic screening was reported to be 2.1% for cervical cancer among women in Accra [14].
3. Sending mobile text messages increased cervical cancer screening rates by over 20% in a one (1) week.pre-post intervention among Korean American Women in Minnesota, USA [22].

*Sing the* Stata command: ssi 0.02 0.20 gave the estimated sample size for two-sample comparison of proportions in an RCT.

Null hypothesis: p1 = p2, where:

- p1 is the proportion of women in the control population (standard practice) who will have had a pap smear test and/or mammogram done by the end of the study. Where p1 = 0.020
- p2 is the proportion of women in the intervention population (mHealth app) who will have done a pap smear test or received a mammogram as a result of the intervention. Where p2 = 0.200
  - power = 0.80
  - alpha = 0.050 (two-sided)

The estimated required sample size (per group) was 44. Allowing for a 20% attrition rate and/or incompletion of questionnaires, the estimated minimum sample size per group became ~ 53 per group.

For the qualitative component, we planned for a maximum of 5 FGDs with about 5–8 women per group. This number of FGDs was deemed sufficient to reach saturation based on evidence by Guest et al. [30] which revealed that more than 90% of all themes were discoverable within three to six focus groups. The study was conducted in four of the 16 administrative units in the Greater Accra Region (GAR) of Ghana [31], including Tema Metropolis, Ashaiman municipality, Accra Sub metropolitan areas, and La Nkwantanang Madina municipality. Women aged between 18 and 39 years who had never taken a cervical cancer test (Pap smear, VIA/VILI, or HPV test), and residents in the four study sites were invited to participate in the study if they (a) possessed a mobile phone to receive SMS text messages and (b) were able to read messages on their mobile phones. The study sample was restricted to women ages 18–39 because we were interested in the screening practices of young adult women of reproductive age as they would likely be at high risk for the outcome of interest as compared to older adult women. In addition, evidence from the ICO/IARC Information Centre on HPV and Cancer, Ghana [5] showed that the estimated coverage of cervical cancer screening in Ghana by age was highest among young adult women; 18–29 years (2.9%) and 30–39 years (4.0%) as compared to women age 40 -49 years (2.7%), 50–49 years (0.2%) and 60–69 years (0.9%).

Ethical approval for the study was obtained from the Institutional Review Board (IRB) of Noguchi Memorial Institute for Medical Research Ethical and Protocol Review Committee (CPN# 011/16-17). All four municipal chief executives for the study sites and managers of banking institutions were engaged and their permission was obtained before data collection. All study methods were carried out following relevant guidelines and regulations.

### Data collection and analyses

#### Qualitative data collection

Five Focus group discussions (FGD) were held between February and May 2017 to obtain perspectives on cervical cancer screening from economically and socially diverse group of women. Each FGD comprised five to eight participants. Participants for two of the FGDs were recruited exclusively from banks and staff working in the La Nkwantanang Madina municipal assembly; i.e., women with “white collar” jobs. For the remaining three FGDs, participants were recruited entirely from communities in La Nkwantanang Madina. This strategy allowed the researchers to obtain diverse perspectives from women with “white collar” jobs and those in the general community. Before each FGD was conducted, written informed consent was obtained from each participant. All FGDs were audio-recorded with the permission of participants and later transcribed verbatim. Each FGD lasted approximately 50 min, with the longest discussion ending at 101 min.

FGDs were moderated by a facilitator who used an interview guide to coordinate the discussions (Additional file 1). The interview guide elicited information on i) awareness of cervical cancer; ii) knowledge about causes or risk factors, prevention, and treatment; iii) awareness of screening tests for cervical cancer; iv) and reasons for which women may or may not want to be screened for cervical cancer. Additional questions regarding participant preference for message contents, length, number of messages sent, at what time messages would be sent, and the duration of time over which the messages would be sent. FGDs participants also discussed the perceived usefulness of SMS messages to encourage uptake of screening, as well as what factors would personally motivate or deter participants from obtaining screening following the receipt of SMS messages.

#### Qualitative data management and analyses

The notes and recordings made during FGDs were transcribed verbatim into Microsoft Word (Microsoft, Redmond, Washington) and then imported into MAX QDA 2018 (VERBI GmbH, Berlin, Germany) and NVivo 11© (QSR Data International, Doncaster, Australia) for data management and thematic analysis. After the initial review, the various views and concepts stemming from the interview guide as well as other issues raised by participants were identified and built into broad themes. These themes provided a framework for coding, and codes were used to organize the texts in the transcripts into nodes. New themes were identified, examined, and cross-referenced to describe and interpret the knowledge women had about cervical cancer, its prevention and screening; treatment of cervical cancer; barriers and facilitators to cervical cancer screening; and the women’s opinions on receiving health messages via SMS.

#### Survey data collection

The baseline surveys were conducted concurrently with the FGDs between February and May 2017. Before survey data collection, field research assistants were trained at the University of Ghana, School of Public Health, after which all data collection tools and procedures were pretested in the Shai Osudoku District in the GAR. As with the FGDs, participants were recruited from bank employees as well as from the general community. Before the survey, information was gathered on the total number of banks in each study area, and banks located on major streets in each study area were listed for recruitment. The study staff contacted bank managers, and administrators of the banks who expressed interest in the study assisted the study team in scheduling meetings with eligible female staff. Those who were willing to participate were screened and recruited.

The initial recruitment strategy sought to recruit one woman per bank to control for clustering, recruit 50% of participants from the banks, and the rest from the communities surrounding the banks. However, banks employed fewer than expected female staff in the eligible age range who were also willing to participate in the study. An amendment to expand the recruitment to include nearby formal institutions (mainly tertiary institutions and municipal health directorates) was made to the protocol, which was subsequently approved by IRB. The remaining participants were subsequently recruited from the Tema Municipal Health Directorate.

To recruit study participants from communities, random starting points were chosen within a 5-km radius from the nearest participating bank. Once a house was identified, a single household was randomly chosen. Within this household, only one woman was invited to participate and screened for eligibility and recruitment. If more than one woman were eligible and willing to participate in the selected household, a single ballot was used to select one participant. Using the main road in the community as a guide, the next third house on the right side of the main road was approached. This systematic sampling procedure was repeated to recruit all community participants.

Survey data were collected using structured questionnaires on Samsung Galaxy Tab 4 tablets [SM-T321 model] (Samsung Electronics, Suwon-si, South Korea) with aided computer-assisted personal interviewing (CAPI). Information collected included basic demographics such as age, place of residence, marital status, education, occupation, and income; basic health history, including smoking status, comorbidities (e.g. hypertension, diabetes, and asthma), family history of cancer, and access to health care. Finally, knowledge and perceptions regarding cervical cancer were assessed, including participant understanding of cervical cancer, willingness to have a Pap smear test, and barriers and facilitators of screening. Additional data were collected on the information that should be included in the text messages, such as causes, prevention, treatment, where to go for a test, cost involved, and what to do if the test result is positive. Willingness to receive SMS messages and to take a Pap smear test or VIA following the receipt of SMS messages were captured, as well as the preferred time, frequency, and duration of the SMS messages (Additional file 2).

#### Survey data management and analysis

The electronic data from the survey were downloaded in Comma Separated Variable (CSV) format and saved as a Microsoft Excel 2007 (Microsoft, Redmond, Washington) before importing into STATA 14.0 (Stata Corporation, College Station, Texas) for further data management. Internal consistency checks were run to ensure the validity and completeness of the data before analyses. Discrete variables were described using frequencies. For continuous variables that were normally distributed, means and standard deviations were employed to estimate summary statistics. For those that were skewed, log transformations were carried out before computing means. To compare the responses of women recruited from banks and those in the communities, Chi-square tests of associations or Fishers' exact tests were performed at a 95% confidence interval and a p-value cutoff of 0.05.

#### Expert stakeholder engagement and development of text library

Stakeholder engagement meetings were held at the School of Public Health, the University of Ghana in June 2017 following the completion of the FGDs and surveys. These stakeholder engagements were designed as a collaborative process to create a short messaging service (SMS) library and to identify the message framework, timing, and frequency using the feedback from FGDs and the survey. Participants of these meetings were specialists/experts from the Ghana Health Service, the Ministry of Health, and the Ghana Atomic Energy Commission who were involved in the management of cervical cancer patients from the Greater Accra Regional, Battor, and the Korle-Bu Teaching Hospitals, respectively. Additionally, five of the investigators participated in the stakeholder meetings.

The initial meeting started with a presentation overviewing the project’s goals and methodology. Findings from the formative research and baseline survey were then discussed, highlighting the barriers and facilitators identified as well as the expectations of the participants on the content and mode of delivery of the text messages. Following these presentations, the group proposed and agreed on the main goal of the text messaging program: to increase uptake of screening among Ghanaian women.

Five communication objectives were also outlined as follows: (1) increase knowledge on cervical cancer; (2) increase knowledge on the importance of early detection; (3) allay fear as a barrier, (4) encourage women to have time for their health, and (5) increase accessibility to screening sites/ provide women with information on screening cost. Text messages were created for each communication objective by phrasing and rephrasing short sentences with actionable words until all participants agreed on the content of each message. Based on these five specific communication objectives, 13 messages were drafted to address cervical cancer knowledge, six messages on the importance of early detection, five messages to allay fear as a barrier, five messages to encourage women to have time for their health, and three messages on where to go for screening and the cost involved (Additional file 3). The framework for the delivery of the text library was also discussed and agreed upon, with the final document specifying the frequency, duration, and Sender ID of the SMS messages.

#### Pretesting of the 32 draft SMS messages

Following the stakeholders’ meeting, the 32 text messages were sent to a new sample of 10 randomly selected women aged 18–39 years from East/West Legon and Madina Municipal for two weeks in July 2017. Over this period, two messages were sent per day, one at 9:00 am and one at 2:00 pm. At the end of the pretest phase, an assessment of the text library and delivery framework were carried out.

The assessment tool (Additional file 4) used was adapted from a tool developed and used in a quasi-experiment that assessed the effectiveness of SMS messages on malaria outpatient case management in the Greater Accra Region [32] which relied on the work of others [27, 33, 34]. The 9 question items in Section A of the tool were used to measure if each of the 32 messages sent was received, read, understood, and interpreted. It also identified any words in the 32 messages that were unclear, difficult to understand, or excessively wordy. Respondents who had indicated willingness to reword any of the text messages were given stickers to reword the unclear messages. The views of the respondents on the relevance of the messages in promoting cervical cancer screening in Ghana were appropriately documented.

The 15 question items in Section B addressed general issues regarding all 32 messages and the delivery framework. The question-item measured how long it took respondents to read the text messages, the time of the day (during working hours, break times, or after the close of business) messages were usually read, experience with the program, most engaging and least engaging aspects of the program, whether there were confusing/irritating aspects of this program, and adequacy of text volume. The assessment tool measured whether the SMS text messages influenced respondents’ views on cervical cancer screening, the acceptability of the SMS promotional messages, and whether they would like the National Non-Communicable Diseases Control Program to send SMS text reminders on cervical cancer screening on their phones in the future. Challenges with mobile network coverage and access to phones during working hours were also documented.

## Results

### Sociodemographic characteristics of the survey participants

Table 1 presents the sociodemographic profile of the 130 participants in the survey. A total of 68 participants were recruited from the communities, with 61 recruited from banks and one respondent who was recruited from Tema Municipal Health Directorate. The mean age of the women was 28.5 years (SD: 5.1). The most predominant ethnic groups represented were Akans and Ga/Adangmes, who constituted the majority (70.8%) of the participants. The Ewes constituted 24.6% of the study population. Almost all the survey participants (126/130) were Christians. A total of 60% (78/130) of the participants were single, while the rest were either married (36.9%) or cohabiting (3.1%). Over 49% (64/130) of the participants had schooling up to the tertiary level. More than half (77/130) of the participants were public servants (77/130). Approximately 12% of the study participants were unemployed; however, about half of the respondents had access to health insurance cards through the National Health Insurance Scheme (NHIS).

**Table 1:** Baseline characteristics of survey participants Survey (N = 130)

Background characteristics	Communities		Banks		Total	%
	(n = 68)	%	(n = 62)	%		
Mean age in years (sd)	28.2 (SD: 5.5)		28.8 (SD: 4.5)		28.5 (SD: 5.1)	
Ethnicity						
Akan	24	35.3	34	54.8	58	44.6
Ga/Dangme	22	32.4	12	19.4	34	26.2
Ewe	18	26.5	14	22.6	32	24.6
Dagbani	0	0	0	0	0	0
Dagaari	0	0	0	0	0	0
Kasem	1	1.5	0	0	1	1
Other (specify)	3	4.4	2	3.2	5	3.9
Total	68	100.1	62	100	130	100.3
Religion						
Christian	66	97.1	60	96.8	126	96.9
Islam	2	2.9	2	3.2	4	3.1
Traditional	0	0	0	0	0	0
None	0	0	0	0	0	0
Other (specify)	0	0	0	0	0	0
Total	68	100	62	100	130	100
Marital status						
Married	20	29.4	28	45.2	48	36.9
Co-habiting	4	5.9	0	0	4	3.1
Divorced	0	0	0	0	0	0
Separated	0	0	0	0	0	0
Single	44	64.7	34	54.8	78	60
Widowed	0	0	0	0	0	0
Total	68	100	62	100	130	100
Level of education						
Primary/middle/JHS	28	41.2	1	1.6	29	22.3
SHS/ O-level / A-level	19	27.9	10	16.1	29	22.3
Vocational/technical	3	4.4	0	0	3	2.3
Tertiary	18	26.5	46	74.2	64	49.2
None	0	0	0	0	0	0
Other (specify)	0	0	5	8.1	5	3.9
Total	68	100	62	100	130	100
Occupation						
Public or civil servant	15	22.1	62	100	77	59.2
Trading	22	32.4	0	0	22	16.9
Artisan	6	8.8	0	0	6	4.6
Apprentice	3	4.4	0	0	3	2.3
Unemployed	15	22.1	0	0	15	11.5
Student	2	2.9	0	0	2	1.5
Other (specify)	5	7.4	0	0	5	3.9
Total	68	100.1	62	100	130	99.9
Access to NHIS*						
No NHIS card	32	47.1	16	25.8	48	36.9
Have NHIS card	36	52.9	46	74.2	82	63.1
Total	68	100	62	100	130	100

*NHIS National Health Insurance Scheme

### Sociodemographic characteristics of participants in the focus group discussions

A total of thirty-two (32) women aged between 21 and 38 years participated in the five (5) FGDs held. One focus group was composed solely of bankers, while participants in the other four groups were engaged in different occupations, including trading, civil/public service, education, and national service (see Table 2).

**Table 2:** Socio-demographics characteristics of participants in the focus group discussions (N = 32)

Groups-participants(n)	Age range (years)	Number of participants (n)
**FGD 1** Service personnel (1) Trader (2) Student (1) Civil/public servant (2)	21–32	6
**FGD 2** Student (4) Trader (1) Civil/public servant (1)	22–35	6
**FGD 3** Teacher (3) Student (3) Religious leader (1)	23–30	7
**FGD 4** Bankers (5)	24–38	5
**FGD 5** Civil/public servant (4) Midwife (1) Service personnel (3)	23–32	8
Total	32	

### Cervical cancer awareness

Cervical cancer awareness was high among the participants surveyed, with 96 (73.9%) having ever heard of cervical cancer. Cervical cancer awareness was higher among women working at the banks (82.3%) than their community counterparts (66.2%). Similar observations were made in the FGDs, with many participants stating they had heard of cervical cancer and were further able to detail that the disease affects only women and occurs in the cervix. They also described it as a change in the cells of the cervix or abnormal growth in the cervix. A few participants had heard of cervical cancer but could supply no further knowledge regarding the disease. The following quotes highlight these points:

It takes place in the cervix as the name makes us understand, cervical means is from the cervix. It is mostly found in women. The cervix is connected around the neck of the womb so I will say cervical cancer is caused by infections or viruses. (p6, 27y/o, teacher, FGD 3).

I have also heard about something about cervical cancer just as she said, anything that has to do with cancer is an unwanted growth that occurs within the cervix of a female. (p5, 27y/o, student, FGD 3).

### Knowledge of causes of cervical cancer

Among the survey participants who had ever heard of cervical cancer, fewer than half were familiar with the causes or risk factors (see Table 3). The majority of the FGD participants stated that the condition was caused by a viral infection and sexual intercourse makes women vulnerable to the infection. Some participants further opined that multiple sexual partners, having unprotected sex, and insertion of herbal preparations and unorthodox medications into the vagina were also causes of cervical cancer. Poor hygiene, contraceptive pills, alcohol and cigarette consumption, multiparity, improper treatment of vagina infections, and sexually transmitted infections (STIs) were also described as possible causes of cervical cancer. The following narratives buttress these opinions:

Mostly it is not the best to say it is contracted through sex because if you have protected sex you will not contract it. It’s only when you have unprotected sex with someone who has the virus then it is likely you might get it. Cancer is an abnormal growth and when the cells are not able to divide then it piles up, so if you indulged in excessive drinking of alcohol, smoking, and then if you also have too many sexual partners you can easily contract such a disease. (p6, 27y/o, teacher, FGD 3).

I don’t know but when growing up people say when you bath with disinfectants like Dettol and those things at the private parts, naturally it cleans itself but people use soaps and insert their finger there so I think those things can also have some effects that can cause so when you do that I think you kill some antigens that fight against external bacteria and all that, you end up killing them so anything at all can cause infections which may lead to… I am not too sure but all those can lead to cancer. (p2, 31y/o, store manager, FGD 1).

What I know is that the more a woman gives birth the higher chance she stands of getting cervical cancer…I think contraceptive pills too…I’m not too sure about this, I think not keeping the place well can also cause it, I mean hygiene…. (p6, 28y/o, receptionist, FGD 2).

**Table 3:** Reported knowledge, attitudes, and practices towards cervical cancer and cervical cancer screening (N = 130)

Characteristics	Communities		Banks		Total	%	Chi-value	*p* value
	No	%	No	%				
Heard of cervical cancer?							4.3	0.04
Yes	45	66.2	51	82.3	96	73.9		
No	23	33.8	11	17.7	34	26.2		
Total	68	100	62	100	130	100.1		
Reported knowledge of causes/risk factors							3.7	0.05
Yes	11	24.4	22	43.1	33	34.4		
No	34	75.6	29	56.9	63	65.6		
Total	45	100	51	100	96	100		
Reported knowledge cervical cancer prevention							0.0637	0.801
Yes	17	37.8	18	35.3	35	36.5		
No	28	62.2	33	64.7	61	63.5		
Total	45	100	51	100	96	100		
Heard of pap smear or VIA test?							2.96	0.086
Yes	13	19.1	20	32.3	33	25.4		
No	55	80.9	42	67.7	97	74.6		
Total	68	100	62	100	130	100		
Necessary to go for pap smear or VIA test?								0.723*
Yes	11	84.6	18	90	29	87.9		
No	0	0	1	5	1	3		
Do Not Know	2	15.4	1	5	3	9.1		
Total	13	100	20	100	33	100		
Intend to do pap smear or VIA test?								0.266*
Yes	8	61.5	17	85	25	75.8		
No	2	15.4	2	10	4	12.1		
Undecided	3	23.1	1	5	4	12.1		
Total		100	20	100	33	100		
Knowledge of place to do pap smear or VIA test?								0.659*
Yes	10	76.9	17	85	27	81.8		
No	3	23.1	3	15	6	18.2		
Total	13	100	20	100	33	100		
Knowledge of cost of pap smear or VIA test?								1.000*
Yes	3	23.1	4	20	7	21.2		
No	10	76.9	16	80	26	78.8		

^*^Fischer's Exact test

### Knowledge about prevention of cervical cancer

From the survey, participants seemed knowledgeable on how to prevent cervical cancer, although slightly more women recruited from the community reported knowing about the preventive measures than their bank counterparts (Table 3).

The FGD participants also knew of preventive measures for cervical cancer  and had varying opinions on how cervical cancer can be prevented. Many felt that education regarding cervical cancer was needed to equip women with the correct information about the disease, its causes, and its prevention. In addition, participants noted that regular check-ups at the hospital were important if the disease was to be detected  early. Safe sex practices such as keeping to a single partner and early treatment of vaginal infections were also suggested. These points are echoed in the narratives below:

Most Ghanaians are not well educated, especially some of the women, they would not know about it, so there should be something like free screening and education in most areas for the women. This will help them to get more knowledge about it to be able to prevent it. (p6, 27y/o, teacher, FGD 3).

Individuals, especially females, should go to the hospital regularly so that it can be detected if you have it so that you can start treating it early before it gets worse. (p3, 24y/o, student, FGD 3).

Being faithful to our partners…you can get it if you have multiple partners so if you stick to one partner, the likelihood that you can get it is low. (p2, 32y/o, banker, FGD 4).

Immunization, good personal hygiene, and eating a well-balanced diet (e.g. avoiding processed foods) were some of the preventive measures also mentioned by the respondents. Participants unanimously agreed that women may prevent cervical cancer by ceasing douching practices and the insertion of herbs and concoctions into the vagina. These opinions are illustrated by the responses below:

We should watch what we eat because some [foods] can also cause cancer. We should check what we eat, especially these canned foods, drinks and all that. (p2, 31y/o, store manager, FGD 1).

I know one can be immunized against cervical cancer at a certain age but am not too sure. (p6, 32y/o, trader, FGD 1).

Yes, mostly the way we keep our private part can even cause infections and sexual intercourse can also cause it, so as you keep the place clean you also have to be careful with who you have sex with, because no matter how neat you keep the place if you don’t take care you can still be affected through sex.” (p6, 28y/o, receptionist, FGD 2).

### Knowledge about treatment of cervical cancer

Knowledge regarding cervical cancer treatment was equal in prevalence among both survey populations, with half stating they knew how cervical cancer treatment was administered (Table 3). While some of the participants in the FGDs did not know of any treatment modalities for cervical cancer, others were able to cite treatment options such as surgery and chemotherapy. FGD participants were also of the opinion that early detection of the condition by a doctor was important. Other ways of treating the disease that was discussed in the FGDs include regular exercise, eating a proper diet, and drinking adequate water. The quotes below illustrate these points:

Looking at the stage that it has reached probably could determine whether you should be given antibiotic, whether you should do surgery, whether it should be chemotherapy. (p5, 31y/o, public servant, FGD 5).

The things we have mentioned so far can help treat it when it is being detected at the early stage, but when it becomes abnormal the only way out is surgery, and even after the surgery it can still grow again so at this stage the surgery alone cannot save you so you have to exercise regularly, taking in more water, eat a good meal and avoid certain things so that your immune system will become strong and fight against the remaining one that wants to grow again, that is what I think. (p5, 27y/o, student, FGD 3).

### Awareness of screening for cervical cancer

Although only one in every four participants surveyed had ever heard about any screening test for cervical cancer, 87.9% of that group thought that it was necessary to go for the tests; 75.8% intended to participate in a screening test if the opportunity arose, and 81.8% knew where they could go for a test. However, only 21.2% of those who had ever heard of a screening test for cervical cancer knew the cost of the test.

A discrepancy in cervical cancer screening knowledge was noted between the participant populations: a greater number of participants who were recruited from the banks reported that they were aware of cervical cancer screenings compared to their community counterparts (32.3% versus 19.1%, respectively). Among those aware of cervical cancer screening, there was little difference seen in the percentage of participants reporting that it was necessary to test for cervical cancer (90% recruited from banks; 84.6% of those recruited from the community. However, this difference was again seen in whether or not participants intended to obtain screening, with 85.0% from the banks reporting that they would, while only 61.5% from the community agreed. More of the bank-recruited participants (81.8%) than from the community (76.9%) reported that they knew where they could obtain Pap smear or VIA tests.

However, where participants had learned about cervical cancer testing methods varied considerably. The health facility was reported as the most popular source of information about cervical cancer screening methods, followed by mass media and the church/mosque (Table 4).

**Table 4:** Source of information on cervical cancer testing methods (N = 33)

Location	Freq	Percent
Health facility (HF)	11	33.3
Pharmacy/drugs store	1	3.0
Church/mosque	4	12.1
School	1	3.0
Mass media	9	27.3
Family/friends	2	6.1
HF and school	1	3.0
HF and mass media	1	3.0
HF and family/friend	2	6.1
Other (can’t remember)	1	3.0
Total	33	100.0

As with the survey respondents, FGD participants were also aware of the need to visit a health facility to obtain screening. FGD participants defined screening as a process that involves the taking of blood and other bodily samples or specimens for examination for specific diseases. Some of the FGD participants were familiar with the Pap smear test to varying degrees. Common sources of information on screening were through doctors, health talks at participant workplaces, or churches. However, among those who had been informed about the need for screening, some participants had declined due to a lack of interest, while others who had received screening stated that the discomfort of the test made them reluctant to be screened again. These factors mentioned are highlighted by the following narratives:

The doctor told me that as a lady you should be having a pap smear once in I think every two years or so. I know a Pap smear will make them detect something. If you are going to have cervical cancer the doctor can detect it and tell you so it is good to have a pap smear. As a lady, you should always be checking out. (p3, 24y/o, administrative assistant, FGD 1).

Some people came to our church some time ago to invite the women and the ladies to come and do free cervical cancer screening test in their private hospital but I didn’t go. (p3, 22y/o, student, FGD 2).

They examine you, to me, it was very uncomfortable. I felt really uncomfortable… the way they did it I was very uncomfortable and I don’t think if you ask me to do it again I would do it. (p2, 31y/o, store manager, FGD 1).

Although most of the respondents deemed cervical cancer screening to be beneficial, they noted that more women would be prepared to access the service if health centers could offer it at no cost. However, participants warned that some might view the testing as poor quality if freely offered. Other participants advocated for free treatment in addition to free screening, as they anticipated screening uptake would not increase without the assurance of free treatment in the event of positive screening.

Ninety-five percent will go once it’s free and it’s about cancer; people will go and even invite their family as well. (p2, 30y/o, teacher, FGD 3).

It’s not just about the screening but the treatment also- if only the screening is free then few people will go because what will be the essence of the free screening if I don’t have the money for treatment if my diagnosis turns out positive? (p5, 27y/o, student, FGD 3).

### Facilitators of cervical cancer screening among women in Accra

Results from the survey demonstrated that the single most important facilitator of cervical cancer screening tests was cervical cancer awareness education (Table 5). Other predominant factors included the desire to know one’s status, a doctor’s recommendation, and showing signs and symptoms associated with cervical cancer. Some women also mentioned family history, high income, availability of time, and having multiple partners as facilitators of cervical cancer screening tests.

**Table 5:** Facilitators of cervical cancer screening tests   (N = 33)

Characteristic	Freq	Percent
Family history	1	3.0
Doctor’s recommendation	3	9.1
Cervical awareness education	12	36.4
Cervical awareness education and showing signs and symptoms of the disease	1	3.0
Cervical awareness and having multiple sexual partners	1	3.0
High income	1	3.0
Showing signs and symptoms	2	6.1
Availability of time	1	3.0
Multiple sexual partners	1	3.0
To know my status	6	18.2
Others	4	12.1
Total	33	100.0

Most of the FGD participants believed that having the test done enabled one to take immediate action following a positive result. Ensuring that women have adequate education just before being screened, providing information on the next steps following a positive screen test, and the provision of free or subsidized tests were all highlighted as steps that would facilitate the screening tests. Despite these opinions, some respondents opined that they would go for the screening only if they began to experience symptoms of cervical cancer. The quotes below elaborate on these points:

The test must be readily available together with the education, so the test should be done right after talking to the person to avoid delay between the education and the actual testing. If you don’t screen them right after the education, by the time you come back next time to screen them their minds would have been polluted by their friends against the screening. (p5, 27y/o, student, FGD 3).

No, but they should know how to put it so that it wouldn’t scare people away, just tell the person about the causes, the effects, and the treatment, tell the truth but do not exaggerate. Let them know if their screening result is negative there is something to prevent it and if it is positive there is a treatment. (p3, 24y/o, student, FGD 3).

I also like to say that one motivating factor could be to working mothers who are free contributors. For instance, in health insurance, as a public servant, you will pay a small amount like GHC5.00. Maybe women in the working class who are SSNIT contributors can be considered, maybe if it won’t be free but maybe some… so that it will give most women the opportunity, because me for instance if I have to go with all my education if I am asked to go and pay GHC100.00 for doing the pap smear, I think today I have to buy tomatoes, I have to buy soap for the house, you know a lot of things keep coming so at the end of the year you might not even be able to go for the screening. So let’s say if it is GHC60.00 and you have to pay GHC40.00, I mean I can part with GHC40.00 to go and pay. (p5, 31y/o, public servant, FGD 5).

### Barriers to cervical cancer screening uptake among women in the study

Survey participants mostly commonly cited fear—either of the disease, the screening procedure, or the outcome—among the obstacles to screening uptake, followed closely by lack of funds. Lack of time was the third most commonly reported barrier to screening. For two-thirds of the respondents (22/33), a combination of lack of time, lack of funds, and fear accounted for the reasons why respondents had not gone for screening (Table 6).

**Table 6:** Barriers to cervical cancer screening uptake among women in the study (N = 33)

Barrier	Freq	Percent
Low-income level	7	21.2
Lack of time	4	12.1
Fear of screening procedure	3	9.1
Fear of cancer	3	9.1
Low-income level and lack of time	2	6.1
Low education on cervical awareness	2	6.1
Fear of screening outcome	2	6.1
Low-income level and fear of cancer	1	3.0
Low education on cervical awareness and fear of screening procedure	1	3.0
Lack of service providers and fear of screening procedure	1	3.0
Other reasons	7	21.2
Total	33	100.0

Similar factors were highlighted by the FGD participants as barriers to cervical cancer screening, including lack of financial resources and fear of having the disease or of possible surgery. Other list factors included distance to the health care centers and time wasted at the test centers due to long queues and bureaucracy. FGD participants also noted that some women would not see the need to go for the screening because they were not ill or had no symptoms. These sentiments are captured in the following narratives:

Some do not want to go for the screening test because they do not have money but if the screening and the treatment are free they will come and screen. But let’s say the screening is free and after the person had screened and she is diagnosed positive, she got to know the treatment is expensive and she can’t afford it, what will happen to the person, knowing that you have it but you don’t have the money to treat it? (p2, 24y/o, sales assistant, FGD 2).

Yeah, proximity is something else and maybe bureaucracy at the facilities, long bureaucracy at the facilities. Delay, unnecessary delays will also discourage one from going. (p5, 31y/o, public servant, FGD 5).

They are scared of knowing they have the disease, even the process and the procedures might scare them so they will prefer not knowing and stay at home. Maybe they will tell you to go for surgery and some people are scared of that so they prefer not knowing at all. (p1, 27y/o, student, FGD 2).

We only go to the hospital when we are sick. That is what has been in our minds since we were born. In the house that we were brought up in, you will not go to the hospital until you are sick even that one unless you are seriously sick. (p2, 32y/o, banker, FGD 4).

Other barriers identified from the FGDs stemmed from both personal and anecdotal experiences and included lack of education on cervical cancer and what the screening entails, poor treatment by the health care providers, fear of stigmatization, and discomfort associated with the procedure. Inadequate counseling at the facilities as well as religious and cultural beliefs requiring a woman to cover up her body was also cited as factors deterring women from accessing screening services.

I think if the person is educated about whatever he or she is going to have the test on, that this is what we are going to do, and these are the symptoms when you have it- then if the person is going to do it the person knows what he or she is up to… In Ghana when you go to the hospital and you want to do a test, they just write for you to go to the lab and do this test. When you get there they just take the paper, look at it, sit, and then they take your sample…Even if it is painful, and if you shout they will say something insulting. I think whoever is going to do the test, should talk to the person. (p2, 31y/o, store manager, FGD 1).

Fear of stigma. Maybe someone has cancer, the stigma that people will see her, she will prefer not to go. (p4, 25y/o, rotation midwife, FGD 5).

Religious beliefs, yes you know some churches do not accept even donation of blood and others. Some too don’t believe in maybe going to open your thighs for someone to see, they think it is a sin. (p1, 32y/o, public servant, FGD 5).

And maybe cultural beliefs too. It could be a factor because some women just think that aside from their husbands and maybe aside from another woman [health personnel] attending to them when it comes to their reproductive organs, the opposite sex shouldn’t see it at all. (p5, 31y/o, public servant, FGD 5).

### Practices of women regarding receiving health information via SMS text messaging

Results from the quantitative survey indicated that almost all participants received text messages on their mobile phones (Table 7). Most of the participants indicated they had ever received health-related text messages on their phones, while a third stated they had never received health-related text messages. Of those who reported receipt of messages on their phones, nearly all usually read the messages.

**Table 7:** Attitudes and practices related to the use of mobile phone and text messages (N = 130)

Characteristics	Communities		Banks		Total	%	Chi-value	*p* value
	No	%	No	%				
Receives text message							0.004	1.000*
Yes	67	98.5	61	98.4	128	98.5		
No	1	1.5	1	1.6	2	1.5		
Total	68	100	62	100	130	100		
Normally reads text messages							2.747	0.246*
Yes	64	95.5	61	100	125	97.7		
No	3	4.5	0	0	3	2.3		
Total	67	100	61	100	128	100		
Ever received text messages on health-related topics							5.381	0.072*
Yes	36	52.9	43	69.4	79	60.8		
No	26	38.2	18	29	44	33.9		
Can't remember	6	8.8	1	1.6	7	5.4		
Total	68	100	62	100	130	100		
Willingness to receive test message on CC testing							1.105	0.477*
Yes	68	100	61	98.4	129	99.2		
No	0	0	1	1.6	1	0.8		
Total	68	100	62	100	130	100		
Willingness to go for CC screening upon receipt of test messages							6.746	0.029*
Yes	62	91.2	61	94.4	123	94.6		
No	0	0	1	1.6	1	0.77		
Undecided	6	8.8	0	0	6	4.6		
Total	68	100	62	100	130	100		
Preferred time of day to receive text messages on CC							14.22	0.010*
Morning	11	16.2	13	21	24	18.6		
Afternoon	4	5.9	5	8.1	9	6.9		
Evening	6	8.8	18	29	24	18.6		
Night	2	2.9	2	3.2	4	3.1		
Anytime	38	55.9	23	37.1	61	46.9		
No suggestion	7	10.3	1	1.6	8	6.2		
Total	68	100	68	62	130	100		
Preferred duration of text messages							1.053	0.907*
Less than 1 month	6	8.8	4	6.5	10	7.7		
1 month	22	32.4	17	27.4	39	30		
2 months	3	4.4	2	3.2	5	3.9		
More than 2 months	22	32.4	24	38.7	46	35.4		
No suggestion	15	22.1	15	24.2	30	23.1		
Total	68	100.0	62	100	130	100.0		
Frequency of text messages per day desired							10.321	0.036*
Once	21	30.9	34	54.8	55	42.3		
Twice	24	35.3	16	25.8	40	30.8		
Thrice	6	8.8	1	1.6	7	5.4		
More than thrice	8	11.8	3	4.8	11	8.5		
No suggestion	9	13.2	8	12.9	17	13.1		
Total	68	100	62	99.9	130	100.0		
Total	68	100	62	100.0	130	100.0		

*Fischer's exact

Many of the FGD participants also indicated that they had received health information by text messaging. The majority of these messages were from mobile network providers. The women also narrated that they unsubscribed from these services because they took up too much battery power, cost money, or were too repetitive as seen in the following narratives:

I have received some before but I can’t remember what it was about, it got to a point I have to unsubscribe it because they were deducting credit for sending me that health information. (p1, 27y/o, student, FGD 2).

Mine was Vodafone helpline, it was a code when you text to that code they will be sending you health tips, I did subscribe but I stopped along the way…because it was taking my battery. (p3, 22y/o, student, FGD 2).

I have subscribed for some before but they kept on repeating the messages, so I also stopped. (p2, 24y/o, sales assistant, FGD 2).

### Willingness to receive health messages on cervical cancer screening

Regarding willingness to receive text messages that encourage women to go for cervical cancer  testing, nearly all quantitative survey participants agreed to receive such text messages, and most said they would subsequently go for cervical screening (Table 7). The FGDs elicited information on the factors that would make women respond positively to a text message promoting cervical cancer screening. It was the consensus that the message should convey that the screening would be carried out either for free or at a reduced cost. FGD participants also suggested that the text should provide information about cervical cancer (with the chance of learning more at the screening center) as well as the closest venue for the screening. Additionally, the messages should also stress the effects of cervical cancer in the service of provoking action. These factors mentioned are highlighted by the quotes below:

It should be something to whet your appetite that is why I am saying that maybe you send me a text message- have you heard about cervical cancer? Do you know its cause? Do you know how it happens?—like you are not giving me answers but you are drawing my attention to something then when I read it I would be, maybe I have not even heard about it before then I am like oh okay. So the next thing if I read all these questions the next thing I will be looking for is the location, is it closer, do I have to spend a lot to go to wherever the screening is taking place? If it is closer then why not ….And, interestingly, everybody is willing to go and check but if you just send me ‘cervical cancer test, SPH room 19, 12 O’clock’, I will look at it and I am like, oh I have heard. (p2, 31y/o, store manager, FGD 1).

The effects, this is what it is, it is caused by this, this is what happens when you are infected. At the end of the day, this is what will happen, if you don’t get treated or if you are infected this is what will happen. And even more, what it is, the cause, and the symptoms. Someone may have even one of the symptoms, [eeii] so am I having it, let me go and do the test. (p8, 28y/o, public servant, FGD 5).

Other ideas proposed by the FGD participants included the use of emboldened texts, catchy phrases, hashtags, and new weekly information to draw the readers’ attention to the campaign and keep them from being bored. Participants also suggested promoting the event in collaboration with a renowned professional or celebrity, as this was capable of evoking interest in the information shared and would further encourage women to be screened.

Because the moment the person sees the notification, the person sees that the cervical cancer is written boldly on it, the person will just tap it and try to read and see what is going on there. (p3, 24y/o, administrative assistant, FGD 1).

Like the way they are saying, a hash-tag like: ‘are you aware’, ‘have you heard’, ‘are you sure’. The teaser is an opener. (p4, 35y/o, banker, FGD 4).

At least every week there should be something new. If you are telling us for instance, this is the number of women dying out of this sickness, if you get to know about the disease you don’t go through this situation. So every week you get to read something new about cervical cancer. (p4, 35y/o, banker, FGD 4).

There should be a renowned (big) person to endorse the message, like a renowned doctor, that will move people to come if not they will see the message alright but they will ignore even though they can read. For example is when they were having this glaucoma exercise, Yvonne Nelson and some other celebrities were involved so it moved people to show interest. (p2, 30y/o, teacher, FGD 3).

#### Negative response to cervical cancer screening text messaging

The FGD discussants anticipated negative responses to the text messages if the opening line were uninteresting, the message came through a short code, or if they felt inundated by the number of messages received. They also opined that the timing of the cervical cancer screening program could coincide with other social engagements, thus preventing the women from taking advantage of the screening opportunity. These opinions are showcased by the following narratives:

I think the first line should be catchy. For me if I receive a text it pops up, if the first line is not anything I am interested in, I just ignore it, I don’t go but if it is a good thing and it catches your attention, let me open my phone and read what else that particular message contains. (p6, 23y/o, service personnel, FGD 5).

But I think if such message should be sent across, it shouldn’t be a short code, it should be a number, now when we get this short code, now are even tired do this and subscribe to this, I just scroll on, I don’t want to do this because I will not subscribe. (p8, 28y/o, public servant, FGD 5).

I think time is a factor. For example, maybe you have a wedding or engagement to go on the same Saturday you are to go for the screening, one will prefer to go for the wedding or the engagement instead of the screening. (p1, 27y/o, student, FGD 2).

Some respondents pointed out that a misleading message where the services offered at the facilities were not as portrayed in the texts could also deter people from accessing the service. Discouraged individuals could also relate their experiences to others thereby leading to feelings of apathy towards the screening programmes.

Sometimes the message does not reflect with the reality. I have personally encountered such a thing before, what they said in the message was not what I saw when I went there and since then it has discouraged me from following up on such messages and since I do not want my friends to go through similar experiences I will discourage them from following up on such messages. (p1, 27y/o, prophetess, FGD 3).

### Preference for text messages in a day

#### Preferred frequency and duration

Survey participants were also asked about the number of text messages on cervical cancer  testing desired per day. Overall, most participants preferred the messages to arrive either once or twice a day (42.3% and 30.8%, respectively), while a few stated they would be amenable to thrice-daily or even more text messages (Table 7). Similarly, the preferred duration of health promotional text messages varied between participants, but over 60% stated that they would prefer “one month” or “more than two months.” A minority of participants preferred “less than one month” or “two months exactly” (see Table 7). However, a considerable number of participants expressed no preference at all.

In the FGDs, the frequency of receiving the text messages was of concern to the women. Many expressed the view that they would not like to receive incessant reminders of the program. While some advocated for a twice-daily reminder, others preferred to receive the texts once or twice a week.

You can send it, twice a day for two weeks in a month, you send twice a day throughout the first week and the same in the last week of the month…or twice a day, two times every week, or twice in a day in the week so that it will not become annoying. (p3, 24y/o, student, FGD 3).

Well it depends, if it is twice a week or once a week, it could be five months, six months or even a year but if it is thrice a week and you send it like five months, I will get bored and feel reluctant to read the subsequent ones because I will feel you are sending the same thing over and over again. (p3, 22y/o, student, FGD 2).

#### Preferred text volume/length

When asked the preferred length of the text messages, the FGD participants suggested a few lines (e.g. fewer than 10 sentences) that occupied not more than two-thirds of the phone screen. They also recommended that the information may be split and sent separately to prevent text-heavy messages that might discourage engagement. The narratives that follow illustrate these points:

Have you heard about it, do you know the causes, do you even know whatever- you can just ask like four questions, four brief questions, if you want to know about it then come to SPH Room 19, Legon campus at this time? It is done, in the end, you will realize that it will not be like ten sentences, or not even up to six. You just frame your question in such a way that a person reads and will be curious. (p2, 31y/o, store manager, FGD 1).

You see we don’t like reading that is one fact we should know, so if it is lengthy, the person will not read. By the time the person even gets to the effects of it, the person has lacked interest and what will boost the person to come forward, the person may not even get there. So it should be just concise. (p1, 32y/o, public servant, FGD 5).

#### Preferred timing of messaging

Survey findings on the preferred time to receive health promotional text messages were mixed, with nearly half stating that any time of the day would work, while the majority of the remaining preferences were mixed between mornings and evenings. A minority of participants preferred the afternoon and night for text messages (Table 7).

FGD participants suggested that times associated with relaxation and leisure were ideal to allow participants time to peruse the messages. For example, they generally agreed that text messages should be sent in the mornings or late evenings to coincide with typical rising times before work or relaxation time after work, respectively. It was also proposed that the messages should come at weekends as well to ensure that women working in different sectors of the economy get the chance to read the message.

Early morning or late evening because when most people wake up from bed the first thing we check is our phones and when you see a message you try to read it but when you are up from the bed you will be busy, and when you are about to sleep too you will like to check through your phone and go through your messages. (p6, 27y/o, teacher, FGD 3).

I share the same idea with her but it should include weekends…because during weekends most people are at home so they will have time to read it. (p6, 28y/o, receptionist, FGD 2).

### Type of information on screening to be included in text messages

Survey respondents exhibited an interest in a wide variety of information related to cervical cancer awareness education (Additional file 5). Generally, this included information ranging from the definitions of cervical cancer to the causes and risk factors, from prevention to treatment, as well as general information regarding screening, including the importance of screening, the cost of testing, where to go, and what to do if the test is positive.

All the women in the FGDs were positive that the text messaging idea would be acceptable to women who receive it, especially if they were literate. Women who could not read would have to depend on their children to explain the content of the text messages. They suggested that a toll-free number for offering advice and answering questions should also be provided in the texts. These views are showcased by the narratives below:

As I said earlier, the cause, the prevention, and the assurance will make it acceptable for someone to go and even invite a friend who didn’t receive the message or is not educated. (p3, 24y/o, student, FGD 3).

For me, I will say yes, in the sense that we all receive messages and anytime you receive a message, you will want to check and see who the message is coming from, whether it’s from your lover or it’s a mobile money alert so there are positive sides. No, because some of our mothers can’t read text messages, they have to wait for their children to come and read it for them so it will be to their disadvantage. (p6, 28y/o, receptionist, FGD 2).

I think if it is a number it should be a toll-free number, not just a phone number, it should be something when you call you won’t pay anything, where you call even for advice., You can call, ‘oh I have seen this- where can I come to your office? Where is your office located?’ I will call and I don’t have to pay anything and they will give me free advice. (p6, 23y/o, service personnel, FGD 5).

However, text messaging alone was not deemed sufficient by the participants, who proposed that the text messages should be complemented with other social media avenues such as WhatsApp and Facebook to improve outreach. In addition, participants advocated for house-to-house education, especially for illiterate women, and the use of visual aids such as videos, posters, and banners to further support the information passed on in the texts.

The messages should include videos and they should be posted on social media like WhatsApp and Facebook. If you see a video of someone suffering from the disease it will scare them to go and check, the message alone is not enough they should also advertise it often on radio and television. (p3, 22y/o, student, FGD 2).

It could also be coupled with something like maybe a post somewhere, maybe a banner pasted around. So you see it and you remember- I got this message some time ago, oh okay they are advertising it here too. (p4, 21y/o, student, FGD 1).

For those who do not have phones and televisions, announcement cars can go there and announce to them about the disease, study groups like the one we are doing now can be organized there for them as well. Community nurses can also help by sensitizing the community about the disease, especially to those who can’t read. In their sensitization, they should do well to mention the causes and the symptoms so that they will rush to the hospital if they see such symptoms. (p2, 24y/o, sales assistant, FGD 2).

### Feedback from the pretest exercise

A total of eight out of the 10 women involved in the pretest exercise were interviewed for a response rate of 80% (Table 8). Messages were reported received by all eight respondents, except for messages 3, 17, 19, 27, 12, and 30. Messages 17, 19, 27, and 12 were received by seven of the respondents, while message 3 was received by only six respondents. One respondent was not sure whether she received the message 30 or not.

**Table 8:** Opinions of respondents on the 32 SMS messages: results from pre-testing of SMS messages (N = 8)

Variable	Frequency	
	(N-8)	Percent
Willingness to receive SMS reminders on CC screening from national non-communicable diseases control program		
Yes	7	87.5
No	1	12.5
Total	8	100
Time of day when text messages are read		
During working hours	2	25.0
After close of work	6	75.0
Appropriateness of timing of messages		
Yes	7	87.5
No	1	12.5
Total	8	100
Adequacy of text volume		
Yes	8	100
Total	8	100
Willingness to receive SMS messages on phones from the Non-Communicable Disease Program		
Yes	7	87.5
No	1	12.5
Total	8	100
Challenges with the mobile network during working hours		
Yes	1	12.5
No	7	87.5
Total	8	100

The text messages were read by all eight respondents, most of whom reported that the messages were clear and easy to understand. However, one respondent did not understand messages 10 and 13. When asked to state the reason why message 10 was not understood the respondent stated that the phrase ‘sexual male, sexual partners’ made the text message confusing, and this was identified as a typographical error. Message 13 was not clearly understood by one respondent because she felt ‘cervical cancer’ was framed as a sexually transmitted infection (STI). All eight respondents reported that texts were of an adequate and concise length, except for messages 10, 11, 22, 23, 26, 27, 29, and 32. For example, one respondent reported excessive length and wordiness in messages 10, 11, and 32. Two of the respondents reported that messages 22, 26, and 29 were slightly wordy.

All eight respondents thought that the 32 test messages were relevant and could increase uptake of cervical cancer screening. Almost all the women (87.5%) said they had read the text messages within one hour of receiving each message. All the eight women interviewed thought that the volume of text messages received was adequate, and seven out of eight women thought that the messages had influenced their views on cervical cancer. When asked about their willingness to receive future SMS text messages, seven (87.5%) out of eight women said they would like to receive SMS text reminders on cervical cancer screening on their phones. Two (25%) out of 8 participants thought that there were some confusing or irritating aspects of the program. One participant (12.5%) felt that the timing of the messages was not appropriate and suggested that the timing of the messages should be changed to 8:30 am to 4:00 pm daily.

## Discussion

This study developed culturally appropriate text messages that could be used to facilitate the uptake of screening services among women working in formal establishments such as the banking sector or general communities. The preferences of respondents regarding the contents and delivery were taken into account in the design of the SMS messaging program. The communication objectives of the SMS messages were derived based on the barriers identified through qualitative and quantitated methods, as Abroms et al. (2015) previously suggested formative research as a necessary first step in developing a messaging health behavior program to provide the basis for understanding population interests and key mechanisms for behavior change [27]. In this study, combining both qualitative and quantitative forms of data collection aided the researchers in obtaining an in-depth understanding of the preferences of respondents regarding SMS messaging programs.

The researchers examined the level of awareness and knowledge regarding the prevention and treatment of cervical cancer among women, finding that nearly three-quarters of respondents had heard of cervical cancer, which suggests a relatively high-level awareness as a type of cancer affecting women. This figure is lower than that obtained from an earlier study conducted among 175 educated women in a similar area in Ghana [35] but higher than those of studies conducted in the metropolitan area of Tamale [36] and a coastal town of Elmina in Ghana [18]. This difference may be due to all participants reporting attainment of least a primary or junior high school level education.

A significant gap exists between the high level of awareness and knowledge regarding the causes, risk factors, prevention, and treatment of the disease. Most women who had ever heard about cervical cancer lacked knowledge about the causes or risk factors. Although the level of knowledge was not assessed, findings from the FGDs suggest that even those who report knowledge may not have access to factual, evidence-based information regarding cervical cancer. Studies conducted in Ghana and other African countries have also reported low awareness of cervical cancer risk factors among women [18, 37–40]. However, a study in Ghana [41] and another in Nigeria [42] found the majority of health workers knew at least one risk factor associated with cervical cancer.

Disparities were also observed among knowledge on the prevention and treatment of cervical cancer. Only about one-third of participants reported that they knew cervical cancer could be prevented, but half of these knew the disease could be treated; these findings were supported by qualitative data obtained in FGD. Other studies in Ghana [18, 39, 43, 44] and elsewhere [45, 46] have also reported a lack of or low awareness of cervical cancer prevention methods among women while recording higher rates of knowledge regarding cervical cancer treatment. One survey among women 15–49 years old in Northeast Ethiopia also found that less than half of respondents knew that cervical cancer is preventable, but more than half of them reported knowing that the disease is treatable [47]. In contrast, a study in Northwest Ethiopia reported that the proportion of women who were aware that cervical cancer is preventable was higher than those who reported that it is curable [48].

It is unclear why some respondents do not know that the disease is preventable, yet report knowing its treatment modalities. The FGD participants in this study discussed surgery and chemotherapy as some of the methods used for treatment even when denying they were aware that cervical cancer is a preventable disease. Poor knowledge of the disease in many African countries could explain this phenomenon [49]. Increasing public awareness and sensitization of key population groups could reduce the gap between awareness and prevention methods, including lifestyle modifications and screening.

Awareness about cervical cancer screening was low among participants in the survey. Only a quarter of participants had heard of a screening test for the illness, with the majority of those having heard about it from the health facility. However, among participants of the focus group discussions in this study, the concept of visiting a health facility and screening for cervical cancer appeared to be generally known, albeit to varying degrees. Recent studies in Ghana [36, 39] and elsewhere in Africa [47, 50, 51] has also reported higher levels of CC screening awareness among participants than in this present study. The most common sources of knowledge were radio/television (30.4%) and doctors/nurses (24.8%), while the least common sources of knowledge were religious bodies (2.5%) and friends and relatives (5.6%).

### Potential facilitators and barriers of screening uptake

Education about cervical cancer, showing signs and symptoms, doctor’s recommendation, and the desire to know one’s status, were found to be the key potential facilitators of cervical cancer screening in the study. Other possible facilitators of screening were the availability of free subsidized tests, high income, and having multiple sexual partners. Several studies in Ghana have also reported education or advice from a health care professional, among other factors, as key enablers of cervical cancer screening in the country [18, 52, 53]. A review of qualitative evidence from across the African continent notes that recognition of signs and symptoms in addition to knowledge of sexual risk factors were important facilitators of cervical cancer screening [54]. Further, a systematic review of published studies in Africa has reported relationships with healthcare providers, social norms, family support, and self-efficacy as key predictors of cervical cancer screening [55]. This present study partly finds agreement with such findings.

Several factors could deter women from availing themselves of cervical cancer screening. Over half (22/33) of the respondents indicated that a combination of lack of time and funds as well as fear of the disease and the screening results accounts for the bulk of the barriers to obtaining screening. These sentiments were also identified in the FGDs. Other potential barriers include fear of stigmatization, difficulty in accessing screening sites, not showing any signs and symptoms, lack of education, and cultural beliefs. Our study findings are consistent with previous studies in Ghana [18, 39, 56, 57]. Several studies across sub-Saharan Africa have reported similar findings of lack of time, lack of knowledge about cervical cancer or screening techniques, as well as fear of the disease and its interventions are the key barriers to widespread adoption of regular screenings [20, 58–60].

The communication objectives focused on addressing gaps that could be targeted through text messaging. These include promoting educational material on disease, its causes, and symptoms, as well as encouraging women to overcome their fear and make time for their health since the benefits of early detection far outweigh the time and resources spent to cure disease later in life. SMS messages, however, cannot be used to address barriers such as difficulty in accessing screening sites or the cost of screening. Because cost remains a significant barrier to cervical cancer screening even among educated women, the National Health Insurance Scheme of Ghana should consider covering the cost of cervical cancer screening among selected high-risk groups to improve the uptake of screening services.

Text messaging through mobile phones may be an appropriate means for addressing the challenges of health literacy related to knowledge gaps and education on cervical cancer. Text messaging has been used successfully to promote health behavior change and health care delivery processes [22, 61, 62]. In the current study, almost all the respondents indicated that they had received the text messages sent to mobile phones. Most women had, in the past, received and read health-related text messages, and were willing to receive SMS promotional messages on cervical cancer. Generally, the respondents were inclined to read messages on the causes, signs, prevention methods, cost of testing, screening sites, and what to do if the test is positive. These concerns were addressed in the creation of the SMS library.

There were notable differences in the preferences of the respondents regarding the frequency, timing, and duration of SMS messages among the qualitative and quantitative data. For example, while some respondents were willing to receive SMS messages early in the mornings, late afternoons, evenings, and weekends, a majority (46.9%: n = 61) were indifferent about the timing of messages. Satisfying the preferences of all the respondents would require a tailored SMS messaging program that permits a developer the flexibility to take into account the individual preferences of end-users. It could, however, be costly [63] and challenging to scale and implement this level of tailoring in low-resource settings. The authors, therefore, designed the SMS messages to suit the preferences of most of the respondents by relying on the data obtained from both the qualitative and quantitative surveys. Respondents generally were against receiving repetitive, long messages as they could cause phone batteries to run low or reduce data availability, and they appeared to prefer an SMS program that would last for two or more months. Results of the pretest show that the selected time, frequency, and duration were acceptable to the respondents. Our findings provide a framework for the content and delivery mode of SMS messages that are likely to meet the preferences of educated women in similar settings elsewhere.

### Study limitations

Since the selection of the sites was determined by the researchers, the results of the study may not be generalizable to other administrative units in the GAR that may be largely rural or peri-urban compared to the metropolitan nature of the study sites. That aside, although bankers constituted almost 50% of the survey participants, they were under-represented in the FGDs, potentially affecting the responses obtained from the FGDs. Again, Women were actively involved in all stages of the study except the Stakeholder's meeting. Involving women in the stakeholder meeting could have potentially further improved the content of the SMS messages because their needs and life situations would have been better understood from their perspective rather than relying on their concerns gleaned from the FGDs and the surveys. Future studies should be designed to address these limitations.

## Conclusion and recommendation

Developing an SMS messaging program that considers the concerns and preferences of a majority of the potential end-users is feasible for Ghanaian women with at least a primary education. Though several factors prevent women from accessing screening services for cervical, barriers such as low levels of education, lack of time, and fear of the screening procedure can be targeted through the communication objectives of SMS messages. The need for health insurance schemes to offset the cost of cervical cancer screening will go a long way to improve uptake, especially among young adult women of reproductive age.

## Supplementary Information

**Additional file 1.** Focus group discussion guide.

**Additional file 2.** Baseline survey questionnaire.

**Additional file 3.** Initial  messages created before pretesting.

**Additional file 4.** Pretest assessment tool.

**Additional file 5.** Type of information to be included in SMS messages.

## Data Availability

The dataset will be made available upon reasonable requests to the corresponding author (aaddo-lartey@ug.edu.gh).

## Abbreviations

CC: Cervical cancer
FGD: Focus group discussion
GAR: Greater Accra Region
IRB: Institutional Review Board
PAP: Papanicolaou
SMS: Short message service
STI: Sexually transmitted infection
VIA: Visual inspection with acetic acid
