# Supplementary material for: Developing a culturally tailored short message service (SMS) intervention for improving the uptake of cervical cancer screening among Ghanaian women in urban communities
Source: BMC Womens Health. 2022 May 10;22:154. doi: 10.1186/s12905-022-01719-9 (PMC9092690; doi:10.1186/s12905-022-01719-9)
Supplement: Supplementary file 1 — Additional file 1. Focus group discussion guide. [file 12905_2022_1719_MOESM1_ESM.doc]

**Supplementary file s1: Focus group discussion guide**

**Objective 1: To assess the knowledge and attitudes of women towards Cervical and Breast cancers**

1. Have you heard of Cervical Cancer/Breast Cancer?

2. What do you think are the causes or Risk factors for Cervical Cancer/Breast Cancer?

3. How do we prevent Cervical Cancer/Breast Cancer?

4. What is the treatment for Cervical Cancer/Breast Cancer?

**Objective 2: To assess the facilitators and barriers to Cervical and Breast cancers screening uptake among women**

1. What is a screening test?

2. Do you know if there is a screening test for Cervical cancer/Breast cancer or not?

3. What are the reasons why women will want to be screened for Cervical Cancer/Breast Cancer?

4. What are the reasons why women will not want to be screened for Cervical Cancer/Breast Cancer?

**Objective 3: To assess the facilitators and barriers to the use of mobile phone technology for screening programs for Cervical and Breast cancers**

1. Have you ever received any health information via SMS on your mobile phone?

2. If you receive an SMS message encouraging you to go for screening tests for Cervical Cancer/Breast Cancer, what factors will make women go for the tests?

3. If you receive an SMS message encouraging you to go for screening tests for Cervical Cancer/Breast Cancer, what factors will make women NOT go for the tests?

4. Perceived Usefulness of such system a system to encourage uptake of screening?

5. What will you consider to be appropriate Information to be sent to you?

6. What will you consider as appropriate timing, Appropriate Duration, Appropriate Volume, Approprate Frequency and Acceptability
