# Supplementary material for: Developing a culturally tailored short message service (SMS) intervention for improving the uptake of cervical cancer screening among Ghanaian women in urban communities
Source: BMC Womens Health. 2022 May 10;22:154. doi: 10.1186/s12905-022-01719-9 (PMC9092690; doi:10.1186/s12905-022-01719-9)
Supplement: Supplementary file 2 — Additional file 2. Baseline survey questionnaire. [file 12905_2022_1719_MOESM2_ESM.docx]

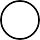

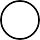

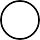

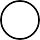

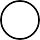

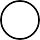

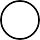


mCBS_BL Cervical Cancer

Eﬀectiveness of a Culturally Tailored Text Messaging Program for Promoting Breast and Cervical Cancer Screening in Accra, Ghana: a Randomized Controlled Trial

DEMOGRAPHIC AND BASELINE CHARACTERISTICS: CERVICAL CANCER

**Respondents District/Municipality/Metropolis**

ASHIAMAN

KPONE KATAMANSO

ACCRA METRO

TEMA METRO

NINGO PRAMPRAM

Respondent 's community of residence

Respondent house number/Address

Survey Type

Baseline

Endline

Respondent ID code

Respondent's Name

*FW should probe for popular name of respondent*

Source of recruitment of respondent


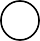


Bank


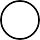


Community

Select the name of the bank

...

Other specify

Branch of Bank

Date of Interview

dd yyyy

FW CODE


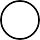


HB


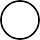


SS


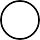


JN


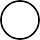


DT

Time Interview Started:

--:-- --

Screening

[q1] Did respondent give consent?


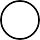


YES


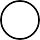


NO

[q1_1] Reasons for refusal

[q2] Have you had any formal education?


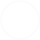


YES


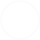


NO

[q3] How old are you in completed years?

[q4] Do you own a personal phone?


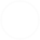


YES


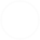


NO

[q5] Are you able to read text messages from your phone?


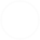


YES


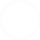


NO

[q6] Have you ever had a Papinicolauo smear Test?


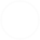


YES


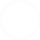


NO

[q7] Do you intend to stay here for the next six (6) months?


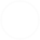


YES


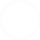


NO

Literacy Test

Ghana is a West African country. There are ten regions in Ghana. The capital city, Accra is located in the Greater Accra region. According to the 2010 population census, the population of the country is about 26,000,000. Ghana is endowed with natural resources which include gold, diamond, oil, cocoa among others.

*FW should let respondent take literacy test*

[q8] Did the respondent pass the literacy test


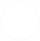


YES


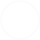


NO


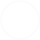


REFUSED

[q9] If respondent refused to take test, what are the reasons for refusal?

**SECTION A: Personal and Family Background Characteristics** FW: Please I would like to ask you a few questions about yourself

[q1] What is your mobile phone number?

*FW: DON'T ENTER THE FIRST ZERO OF THE PHONE NUMBER: If more than 1, FW should document the number the respondent uses regularly*

[q2] How old are you? (in completed years)

*FW: AGE SHOULD BE BEWTEEN 18 AND 39 YEARS*

[q3] What is your marital status?


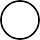


Married


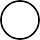


Co-habiting


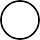


Divorced


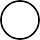


Separated


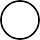


Single


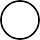


Widowed


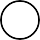


Vocational/Technical


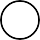


Tertiary


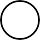


None


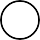


Other (specify)

[q4_oth] Other specify

[q5] What is your occupation?


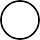


Public/Civil Servant


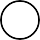


Trading


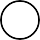


Artisan


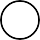


Apprentice


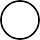


Unemployed


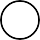


Student


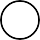


Retired


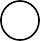


Other (specify)

[q5_oth] Other specify


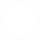


Ghc500.00 -Ghc999.00


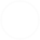


Ghc1000.00 -Ghc2000.00


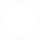


Above Ghc2000


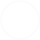


No Income

[q7] What is your ethnicity?


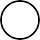


Akan


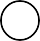


Ga/Dangme


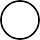


Ewe


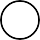


Dagbani


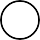


Dagaari


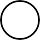


Kasem


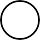


Other (specify)

[q7_oth] Other specify


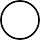


Traditional


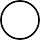


None


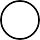


Other (specify)

[q8_oth] Other specify

[q9] Do you have a valid NHIS or any other health insurenace scheme card


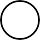


YES


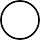


NO

[q10] Have you ever smoked cigarette?


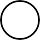


YES


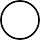


NO

[q11] If yes, do you smoke cigarette currently?


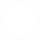


YES


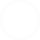


NO

[q12] How often do you smoke?


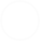


Daily


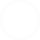


Weekly


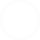


Once in a while


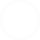


Over 20 sticks per day (heavy)

[q14] Have you ever drunk alcohol?


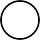


YES


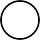


NO

[q15] Do you drink alcohol currently?


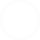


YES


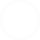


NO

[q16] How often do you drink?


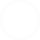


Daily


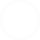


Weekly


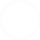


Once in a while

[q17] Have you ever taken oral contraceptives?


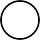


YES


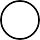


NO

[q17_1] if yes, are you currently taking oral contraceptives?

*Caution: FW should not ask obviously pregnant women but instead observe and answer*


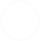


YES


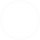


NO

[q18] Mention the names of the oral contraceptive pills you have ever used.

Secure oral Pill

Lydia Oral Pill

Lydia Daphne

Lydia Post Pill

Cytotec

Today contraceptive

Postinor 2

Other (specify)

[a18_oth] Other specify

[19] How old were you when you had your ﬁrst menses (menarche)?

*Please enter in years*


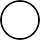


Less than 12 years


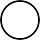


12 - 14 years


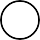


15 and over years


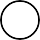


Can't remember

[q20] Do you suﬀer from any of these Chronic illnesses?

[q20_1] Hypertension [q20_2] Diabetes Melitus

[q20_3] Hyper-Cholesterolemia

[q20_4] Asthma [q20_5] Other

[q20_oth] Other Chronic illnesses? Please specify

YES NO DO NOT KNOW


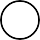

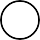

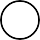

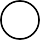

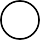

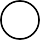

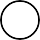

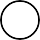

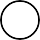

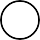

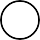

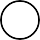

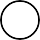

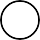

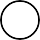


DO NOT WANT TO DISCLOSE

[q21] Does any close relation suﬀer from any of these Chronic illnesses?

[q21_1] Hypertension [q21_2] Diabetes Melitus

[q21_3] Hyper-Cholesterolemia

[q21_4] Asthma [q21_5] Other

[a21_oth] Other Chronic illnesses? Please specify

YES NO DO NOT KNOW

DO NOT WANT TO DISCLOSE

[q22] Is there a history of Cancer in your family?

YES

NO

DO NOT KNOW

DO NOT WANT TO DISCLOSE

[q23] If yes, what type of Cancer?

Head & Neck

Chest

Abdominal

Pelvic

Skeletal

Skin

Muscle

Don’t know

Don’t want to disclose

Other (specify)

[q23_oth] Other specify

District hospital

Polyclinic

Health centre

Private hospital

Pharmacy/chemical shop

Police/Military Hospital

Other (specify)

[q24] Other (specify)…………………

[q25] How many times did you go for a Doctor's visit last year?

None

Once

Twice

Thrice

More than thrice

Don't know/Cannot remember

**SECTION B: Knowledge, Attitude and Practices towards Breast Cancer** FW: Please I would like to ask you a few questions about Cervical cancer

[q27] If yes, do you know about the causes/risk factors?

YES

NO

[b28] If yes, (specify)…………………

[q29] Do you know Cervical cancer can be prevented?

YES

NO

[q30] If yes, how can it be prevented? (specify)…………………………

SECTION C: Screening test

FW: Please I would like to ask you a few questions about cervical cancer screening test

[q31] Have you ever heard of Papanicolauo (Pap) Smear test or Visual Inspection with Acetic acid (VIA) test?

YES

NO

[q32] If yes to q31, what did you hear about Pap smear or VIA test ?

[q32] FW to indicate if apprpriate or Inappropriate

Appropriate

Inappropriate

Church/Mosque

School

Television

Radio

Newspaper

Internet

Family/Friends

Other (specify)

[q33] Other (specify)…………………

[q34] If yes to q31, who gave you the information about Pap smear test or VIA test?

Health Worker

Health Researcher

Other (specify)

[q34] Other (specify)…………………

DO NOT KNOW

[q36] What will make you go for the Pap smear test or VIA test?

Family History of Cervix cancer

Doctor recommendation

Cervix Awareness education

High income level

Nearness to Service provider

Other (specify)

[q36] Other (specify)…………………

Low education on Cervix awareness

Lack of awareness on test

Lack of Doctor referral

Lack of time

Fear of cancer

Fear of screening procedure

Fear of screening outcome

Fear of Cervix surgery

Fear of neglect/abandonment

Fear of death

Low priority of individual's health

Lack of consent from signiﬁcant others

Other (specify)

[q37] Other (specify)…………………

UNDECIDED

[q39] Do you know where to go for a Pap smear test or VIA test?

YES

NO

[q40] Do you know how much it cost to do a Pap smear test or VIA test?

YES

NO

[q41] If yes to 40, Please state the cost.

SECTION D: Mobile phone Text Messaging on Cervix Cancer

FW: Please I would like to ask you a few questions on receiving health information messages about Cervical Cancer on your mobile phone)

[q42] Do you receive text message on your mobile phone?

YES

NO

[q43] If yes, do you normally read these text messages?

YES

NO

CAN'T REMEMBER

[q45] Would you want to receive text messages on your mobile phone encouraging you to go for Pap smear test or VIA test?

YES

NO

[q46] If we decide to send you text messages on your mobile phone encouraging you to go for a Pap smear test or VIA test, what information on cervical cancer/screening would you expect to be included in the text messages?

What Cervix cancer is

Causes/Risk factors

Prevention

Treatment

Importance of Pap smear/VIA

Where to go for Pap smear/VIA

Cost of Pap smear/VIA

What to do if Pap smear/VIA is positive

Other (specify)

[q46] Other (specify)…………………

[q47] If you receive text messages on your mobile phone informing you about the need for you to go for Pap smear test or VIA test, would you be willing to go?

YES

NO

UNDECIDED

[q48] At what time of the day would you prefer to receive these text messages?

Morning

Afternoon

Evening

Night

Anytime of the day

No Suggestion

[q49] How long would you want to receive these text messages?

Less than 1 month

1 month

2 months

More than 2 months

No Suggestion

[q50] How many times in a day would you like to receive these text messages?

Once

Twice

Thrice

More than thrice

No Suggestion

[q51] Apart from SMS texts, what other ‘private’ medium/media would you like to receive health information on?

Whatsapp

Imo

Viber

Telegram

Email

Facebook

Instagram

Twitter

None

Other (specify)

[q51] Other (specify)…………………

WE HAVE NOW COME TO THE END OF THIS SURVEY. THANK YOU VERY MUCH FOR PARTICIPATING!

INTWER: ENTER FIELD NOTES/COMMENTS

INTWER: TAKE GPS

latitude (x.y °) longitude (x.y °) altitude (m) accuracy (m)
