# Supplementary material for: Developing a culturally tailored short message service (SMS) intervention for improving the uptake of cervical cancer screening among Ghanaian women in urban communities
Source: BMC Womens Health. 2022 May 10;22:154. doi: 10.1186/s12905-022-01719-9 (PMC9092690; doi:10.1186/s12905-022-01719-9)
Supplement: Supplementary file 3 — Additional file 3. Initial messages created before pretesting. [file 12905_2022_1719_MOESM3_ESM.docx]

**Additional file s3: Initial draft 32 messages created before pre-testing**

Knowledge of Cervical cancer

- 1. Have you heard? Cervical Cancer is the second most common cancer in women in Ghana. Go for screening!
- 2. Cervical cancer is preventable. Go for screening!
- 3. About 3000 women get cervical cancer every year in Ghana. Go for screening!
- 4. Report any abnormal vaginal bleeding at a hospital.
- 5. If you any abnormal vaginal discharge. Ask a Doctor. It may not be Cancer!
- 6. Persistent vaginal discharge following treatment requires further testing. Go for screening!
- 7. Cervical cancer can be cured if detected and treated early. Go for screening!
- 8. Reporting late to the Doctor makes treatment difficult.
- 9. Cervical cancer is not a spiritual disease. It can be treated in a hospital.
- 10. Do you know that multiple current and lifetime sexual partners increase your risk of getting cervical cancer?
- 11. Do you know that delaying initiation of first sexual intercourse till you are older than 20 reduces your risk of getting cervical cancer?
- 12. If you have ever had a sexually transmitted infection, you are at risk of getting cervical cancer
- 13. Condom is not complete protection from cervical cancer

Knowledge on importance of early detection

- 1. Do not let Cervical cancer take you away from your loved ones. Have you gone for screening yet?
- 2. Cervical cancer should not kill you. You kill Cervical cancer! Go for screening!
- 3. Your life can be saved if Cervical cancer is detected early. Go for screening
- 4. Do not let Cervical cancer kill your dreams. Go for screening!
- 5. Ghana needs you alive. Go for cervical cancer screening!
- 6. Early detection of Cervical cancer reduces the cost of treatment. Go for screening!

Allay Fear as a barrier

- 1. Cervical cancer screening is not harmful! Go for cervical cancer screening
- 2. Cervical cancer is curable when detected early. Go for cervical cancer screening
- 3. ? out of every? women who have their cervix checked regularly are saved from cervical cancer and prevent death. Go for screening!
- 4. ? out of every? women who have early detection and treatment for cervical cancer are still alive after 5 years. Go for screening!
- 5. Having Cervical cancer is not a death sentence. It can be treated. Go for screening!

Encourage to have time for their health

- 1. Your Health is your Wealth. Make time for your Health. Go for cervical cancer screening!
- 2. Have time for Cervical cancer screening today. Save your life tomorrow. Spend time on your health. It is not time wasted.
- 3. Take time off your busy schedules and go for cervical cancer screening. A short time spent on cervical cancer screening saves a long stay at the hospital for treatment.
- 4. Have you had your cervical cancer screening? Make time for it today!
- 5. Cervical cancer screening does not take long. You spend less time testing for Cervical cancer than you spend at the hairdresser’s

Accessibility to screening sites/cost

- 1. Cervical cancer screening is available in Ghana.

You can visit any of the following facilities;

1. Tema General Hospital,

2. Narh-Bita Hospital,

3. Korle-Bu Teaching Hospital,

4. Ridge Hospital,

- 2. The cost of cervical cancer screening is far less than the cost of treatment.
- 3. It is better to spend your pocket money today on cervical cancer screening than to spend your life savings on treatment tomorrow#
