# Supplementary material for: Developing a culturally tailored short message service (SMS) intervention for improving the uptake of cervical cancer screening among Ghanaian women in urban communities
Source: BMC Womens Health. 2022 May 10;22:154. doi: 10.1186/s12905-022-01719-9 (PMC9092690; doi:10.1186/s12905-022-01719-9)
Supplement: Supplementary file 4 — Additional file 4. Pretest assessment tool. [file 12905_2022_1719_MOESM4_ESM.docx]

sms_y_cervical

ASSESSMENT OF SMS TEXT MESSAGES

Eﬀectiveness of a Culturally Tailored Text Messaging Program for Promoting Breast and Cervical Cancer Screening in Accra, Ghana: a Randomized Controlled Trial

Form Number

Date of Interview

yyyy-mm-dd

SURVEY TYPE: CERVICAL CANCER

Time Interview Started:

hh:mm

FW CODE


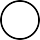
 HB
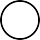
 SS
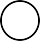
 JN
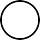
 DT

Respondent's Name

*FW should probe for popular name of respondent*

Respondent category


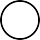
 COMMUNITY
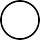
 ASSEMBLY/BANK

My name is I am with a team of researchers working on the study that seeks to assess whether SMS text messages can

be used to improve upon uptake for breast and cervical cancer screening in Greater Accra, Ghana.

I would like to ask you a few questions about the SMS text Messages you received from UG-SPH. Please note that the information you provide would be used for the purposes of research work only. Can we start?

Respondent ID code

Message CC1

"Have you heard? Cervical Cancer is the second most common cancer in women in Ghana. Go for screening!".

*( FW should read the text messages or Show Message Log to Respondent)*

[Q1] Did you ever receive this message?


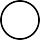
 YES
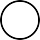
 NO


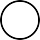
 NOT SURE
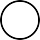
 CAN'T RECALL

[Q2] Did you ever read this message?

*If Respondent did not read the text messages, let him read the text message and continue with questions*


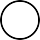
 YES
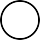
 NO


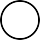
 NOT SURE
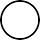
 CAN'T RECALL

[Q3] Was the text message easy to understand/clear?


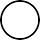
 YES
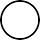
 NO


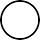
 NOT SURE
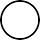
 CAN'T RECALL

[Q4] Could you please indicate why this text message is diﬃcult or not easy to understand/ unclear?

*NA--999*

[Q5] In your own few words can you tell me how you understood this message?

*NA--999*

[Q6] Was this SMS text too long/wordy?


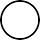
 YES
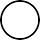
 NO


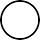
 NOT SURE
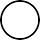
 SOMEHOW
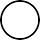
 DON'T KNOW

[Q7] Would you like to reword/rewrite the text message?


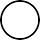

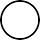
 YES
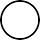
 NO

NOT APPLICABLE

[Q8] How would you want to reword this message?

*(provide a sticker for rewording or write in the space below) NA---999*

[Q9] Do you think that this SMS text would be relevant in increasing uptake of Breast cancer screening?


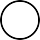
 YES
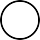
 NO

Message CC2

"Cervical cancer is preventable. Go for screening!"

*( FW should read the text messages or Show Message Log to Respondent)*

[Q1] Did you ever receive this message?


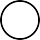
 YES
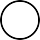
 NO


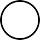
 NOT SURE
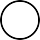
 CAN'T RECALL

[Q2] Did you ever read this message?

*If Respondent did not read the text messages, let him read the text message and continue with questions*


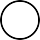
 YES
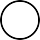
 NO


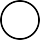
 NOT SURE
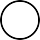
 CAN'T RECALL

[Q3] Was the text message easy to understand/clear?


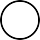
 YES
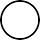
 NO


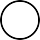
 NOT SURE
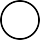
 CAN'T RECALL

[Q4] Could you please indicate why this text message is diﬃcult or not easy to understand/ unclear?

*NA--999*

[Q5] In your own few words can you tell me how you understood this message?

*NA--999*

[Q6] Was this SMS text too long/wordy?


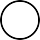
 YES
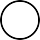
 NO


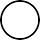
 NOT SURE
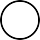
 SOMEHOW
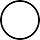
 DON'T KNOW

[Q7] Would you like to reword/rewrite the text message?


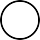
 YES
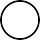
 NO


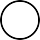
 NOT APPLICABLE

[Q8] How would you want to reword this message?

*(provide a sticker for rewording or write in the space below) NA---999*

[Q9] Do you think that this SMS text would be relevant in increasing uptake of Breast cancer screening?


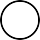
 YES
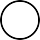
 NO

Message CC3

"About 3000 women get cervical cancer every year in Ghana. Go for screening!"

*( FW should read the text messages or Show Message Log to Respondent)*

[Q1] Did you ever receive this message?


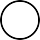
 YES
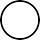
 NO


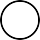
 NOT SURE
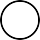
 CAN'T RECALL

[Q2] Did you ever read this message?

*If Respondent did not read the text messages, let him read the text message and continue with questions*


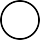
 YES
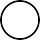
 NO


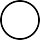

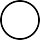
 NOT SURE CAN'T RECALL


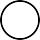
 YES
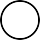
 NO


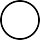

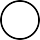
 NOT SURE CAN'T RECALL

[Q4] Could you please indicate why this text message is diﬃcult or not easy to understand/ unclear?

*NA--999*

[Q5] In your own few words can you tell me how you understood this message?

*NA--999*

[Q6] Was this SMS text too long/wordy?


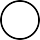
 YES
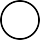
 NO


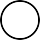
 NOT SURE
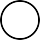
 SOMEHOW
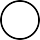
 DON'T KNOW

[Q7] Would you like to reword/rewrite the text message?


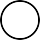
 YES
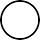
 NO


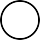
 NOT APPLICABLE

[Q8] How would you want to reword this message?

*(provide a sticker for rewording or write in the space below) NA---999*

[Q9] Do you think that this SMS text would be relevant in increasing uptake of Breast cancer screening?


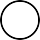
 YES
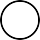
 NO

Message CC4

"Report any abnormal vaginal bleeding at a hospital."

*( FW should read the text messages or Show Message Log to Respondent)*

[Q1] Did you ever receive this message?


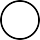
 YES
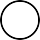
 NO


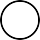
 NOT SURE
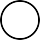
 CAN'T RECALL

[Q2] Did you ever read this message?

*If Respondent did not read the text messages, let him read the text message and continue with questions*


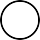
 YES
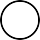
 NO


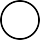
 NOT SURE
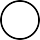
 CAN'T RECALL

[Q3] Was the text message easy to understand/clear?


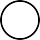
 YES
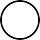
 NO


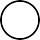
 NOT SURE
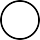
 CAN'T RECALL

[Q4] Could you please indicate why this text message is diﬃcult or not easy to understand/ unclear?

*NA--999*

[Q5] In your own few words can you tell me how you understood this message?

*NA--999*

[Q6] Was this SMS text too long/wordy?


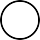
 YES
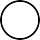
 NO


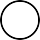
 NOT SURE
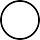
 SOMEHOW
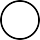
 DON'T KNOW

[Q7] Would you like to reword/rewrite the text message?


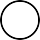

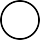
 YES
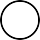
 NO

NOT APPLICABLE

[Q8] How would you want to reword this message?

*(provide a sticker for rewording or write in the space below) NA---999*

[Q9] Do you think that this SMS text would be relevant in increasing uptake of Breast cancer screening?


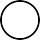
 YES
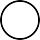
 NO

Message CC5

"If you any abnormal vaginal discharge. Ask a Doctor. It may not be Cancer!"

*( FW should read the text messages or Show Message Log to Respondent)*

[Q1] Did you ever receive this message?


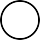
 YES
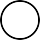
 NO


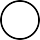
 NOT SURE
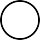
 CAN'T RECALL

[Q2] Did you ever read this message?

*If Respondent did not read the text messages, let him read the text message and continue with questions*


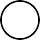
 YES
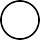
 NO

NOT SURE CAN'T RECALL

[Q3] Was the text message easy to understand/clear?

YES NO

NOT SURE CAN'T RECALL

[Q4] Could you please indicate why this text message is diﬃcult or not easy to understand/ unclear?

*NA--999*

[Q5] In your own few words can you tell me how you understood this message?

*NA--999*

[Q6] Was this SMS text too long/wordy?

YES NO

NOT SURE SOMEHOW DON'T KNOW

[Q7] Would you like to reword/rewrite the text message?

YES NO

NOT APPLICABLE

[Q8] How would you want to reword this message?

*(provide a sticker for rewording or write in the space below) NA---999*

[Q9] Do you think that this SMS text would be relevant in increasing uptake of Breast cancer screening?

YES NO

Message CC6

"Persistent vaginal discharge following treatment requires further testing. Go for screening!"

*( FW should read the text messages or Show Message Log to Respondent)*

[Q1] Did you ever receive this message?

YES NO

NOT SURE CAN'T RECALL

[Q2] Did you ever read this message?

*If Respondent did not read the text messages, let him read the text message and continue with questions*

YES NO

NOT SURE CAN'T RECALL

YES NO

NOT SURE CAN'T RECALL

[Q4] Could you please indicate why this text message is diﬃcult or not easy to understand/ unclear?

*NA--999*

[Q5] In your own few words can you tell me how you understood this message?

*NA--999*

[Q6] Was this SMS text too long/wordy?

YES NO

NOT SURE SOMEHOW DON'T KNOW

[Q7] Would you like to reword/rewrite the text message?

YES NO

NOT APPLICABLE

[Q8] How would you want to reword this message?

*(provide a sticker for rewording or write in the space below) NA---999*

[Q9] Do you think that this SMS text would be relevant in increasing uptake of Breast cancer screening?

YES NO

Message CC7

"Cervical cancer can be cured if detected and treated early. Go for screening!"

*( FW should read the text messages or Show Message Log to Respondent)*

[Q1] Did you ever receive this message?

YES NO

NOT SURE CAN'T RECALL

[Q2] Did you ever read this message?

*If Respondent did not read the text messages, let him read the text message and continue with questions*

YES NO

NOT SURE CAN'T RECALL

[Q3] Was the text message easy to understand/clear?

YES NO

NOT SURE CAN'T RECALL

[Q4] Could you please indicate why this text message is diﬃcult or not easy to understand/ unclear?

*NA--999*

[Q5] In your own few words can you tell me how you understood this message?

*NA--999*

[Q6] Was this SMS text too long/wordy?

YES NO

NOT SURE SOMEHOW DON'T KNOW

[Q7] Would you like to reword/rewrite the text message?

YES NO

NOT APPLICABLE

[Q8] How would you want to reword this message?

*(provide a sticker for rewording or write in the space below) NA---999*

[Q9] Do you think that this SMS text would be relevant in increasing uptake of Breast cancer screening?

YES NO

Message CC8

"Reporting late to the Doctor makes treatment diﬃcult. Go for screening!"

*( FW should read the text messages or Show Message Log to Respondent)*

[Q1] Did you ever receive this message?

YES NO

NOT SURE CAN'T RECALL

[Q2] Did you ever read this message?

*If Respondent did not read the text messages, let him read the text message and continue with questions*

YES NO

NOT SURE CAN'T RECALL

[Q3] Was the text message easy to understand/clear?

YES NO

NOT SURE CAN'T RECALL

[Q4] Could you please indicate why this text message is diﬃcult or not easy to understand/ unclear?

*NA--999*

[Q5] In your own few words can you tell me how you understood this message?

*NA--999*

[Q6] Was this SMS text too long/wordy?

YES NO

NOT SURE SOMEHOW DON'T KNOW

[Q7] Would you like to reword/rewrite the text message?

YES NO

NOT APPLICABLE

[Q8] How would you want to reword this message?

*(provide a sticker for rewording or write in the space below) NA---999*

[Q9] Do you think that this SMS text would be relevant in increasing uptake of Breast cancer screening?

YES NO

Message CC9

"Cervical cancer is not a spiritual disease. It can be treated in a hospital. Go for screening!"

*( FW should read the text messages or Show Message Log to Respondent)*

[Q1] Did you ever receive this message?

YES NO

NOT SURE CAN'T RECALL

[Q2] Did you ever read this message?

*If Respondent did not read the text messages, let him read the text message and continue with questions*

YES NO

NOT SURE CAN'T RECALL

YES NO

NOT SURE CAN'T RECALL

[Q4] Could you please indicate why this text message is diﬃcult or not easy to understand/ unclear?

*NA--999*

[Q5] In your own few words can you tell me how you understood this message?

*NA--999*

[Q6] Was this SMS text too long/wordy?

YES NO

NOT SURE SOMEHOW DON'T KNOW

[Q7] Would you like to reword/rewrite the text message?

YES NO

NOT APPLICABLE

[Q8] How would you want to reword this message?

*(provide a sticker for rewording or write in the space below) NA---999*

[Q9] Do you think that this SMS text would be relevant in increasing uptake of Breast cancer screening?

YES NO

Message CC10

"Do you know that multiple current and life time sexual male sexual partners increases your risk of getting cervical cancer? Go for screening!"

*( FW should read the text messages or Show Message Log to Respondent)*

[Q1] Did you ever receive this message?

YES NO

NOT SURE CAN'T RECALL

[Q2] Did you ever read this message?

*If Respondent did not read the text messages, let him read the text message and continue with questions*

YES NO

NOT SURE CAN'T RECALL

[Q3] Was the text message easy to understand/clear?

YES NO

NOT SURE CAN'T RECALL

[Q4] Could you please indicate why this text message is diﬃcult or not easy to understand/ unclear?

*NA--999*

[Q5] In your own few words can you tell me how you understood this message?

*NA--999*

[Q6] Was this SMS text too long/wordy?

YES NO

NOT SURE SOMEHOW DON'T KNOW

[Q7] Would you like to reword/rewrite the text message?

YES NO

NOT APPLICABLE

[Q8] How would you want to reword this message?

*(provide a sticker for rewording or write in the space below) NA---999*

[Q9] Do you think that this SMS text would be relevant in increasing uptake of Breast cancer screening?

YES NO

Message CC11

"Do you know that early initiation of ﬁrst sexual intercourse before the age of 20 increases your risk of getting cervical cancer?"

*( FW should read the text messages or Show Message Log to Respondent)*

[Q1] Did you ever receive this message?

YES NO

NOT SURE CAN'T RECALL

[Q2] Did you ever read this message?

*If Respondent did not read the text messages, let him read the text message and continue with questions*

YES NO

NOT SURE CAN'T RECALL

[Q3] Was the text message easy to understand/clear?

YES NO

NOT SURE CAN'T RECALL

[Q4] Could you please indicate why this text message is diﬃcult or not easy to understand/ unclear?

*NA--999*

[Q5] In your own few words can you tell me how you understood this message?

*NA--999*

[Q6] Was this SMS text too long/wordy?

YES NO

NOT SURE SOMEHOW DON'T KNOW

[Q7] Would you like to reword/rewrite the text message?

YES NO

NOT APPLICABLE

[Q8] How would you want to reword this message?

*(provide a sticker for rewording or write in the space below) NA---999*

[Q9] Do you think that this SMS text would be relevant in increasing uptake of Breast cancer screening?

YES NO

Message CC12

"If you have ever had a sexually transmitted infection, you are at risk of getting cervical cancer. Go for screening!"

*( FW should read the text messages or Show Message Log to Respondent)*

[Q1] Did you ever receive this message?

YES NO

NOT SURE CAN'T RECALL

[Q2] Did you ever read this message?

*If Respondent did not read the text messages, let him read the text message and continue with questions*

YES NO

NOT SURE CAN'T RECALL

YES NO

NOT SURE CAN'T RECALL

[Q4] Could you please indicate why this text message is diﬃcult or not easy to understand/ unclear?

*NA--999*

[Q5] In your own few words can you tell me how you understood this message?

*NA--999*

[Q6] Was this SMS text too long/wordy?

YES NO

NOT SURE SOMEHOW DON'T KNOW

[Q7] Would you like to reword/rewrite the text message?

YES NO

NOT APPLICABLE

[Q8] How would you want to reword this message?

*(provide a sticker for rewording or write in the space below) NA---999*

[Q9] Do you think that this SMS text would be relevant in increasing uptake of Breast cancer screening?

YES NO

Message CC13

"Condom is not a complete protection from cervical cancer. Go for screening!"

*( FW should read the text messages or Show Message Log to Respondent)*

[Q1] Did you ever receive this message?

YES NO

NOT SURE CAN'T RECALL

[Q2] Did you ever read this message?

*If Respondent did not read the text messages, let him read the text message and continue with questions*

YES NO

NOT SURE CAN'T RECALL

[Q3] Was the text message easy to understand/clear?

YES NO

NOT SURE CAN'T RECALL

[Q4] Could you please indicate why this text message is diﬃcult or not easy to understand/ unclear?

*NA--999*

[Q5] In your own few words can you tell me how you understood this message?

*NA--999*

[Q6] Was this SMS text too long/wordy?

YES NO

NOT SURE SOMEHOW DON'T KNOW

[Q7] Would you like to reword/rewrite the text message?

YES NO

NOT APPLICABLE

[Q8] How would you want to reword this message?

*(provide a sticker for rewording or write in the space below) NA---999*

[Q9] Do you think that this SMS text would be relevant in increasing uptake of Breast cancer screening?

YES NO

Message CC14

"Do not let Cervical cancer take you away from your loved ones. Have you gone for screening yet?"

*( FW should read the text messages or Show Message Log to Respondent)*

[Q1] Did you ever receive this message?

YES NO

NOT SURE CAN'T RECALL

[Q2] Did you ever read this message?

*If Respondent did not read the text messages, let him read the text message and continue with questions*

YES NO

NOT SURE CAN'T RECALL

[Q3] Was the text message easy to understand/clear?

YES NO

NOT SURE CAN'T RECALL

[Q4] Could you please indicate why this text message is diﬃcult or not easy to understand/ unclear?

*NA--999*

[Q5] In your own few words can you tell me how you understood this message?

*NA--999*

[Q6] Was this SMS text too long/wordy?

YES NO

NOT SURE SOMEHOW DON'T KNOW

[Q7] Would you like to reword/rewrite the text message?

YES NO

NOT APPLICABLE

[Q8] How would you want to reword this message?

*(provide a sticker for rewording or write in the space below) NA---999*

[Q9] Do you think that this SMS text would be relevant in increasing uptake of Breast cancer screening?

YES NO

Message CC15

"Cervical cancer should not kill you. You kill Cervical cancer! Go for screening!"

*( FW should read the text messages or Show Message Log to Respondent)*

[Q1] Did you ever receive this message?

YES NO

NOT SURE CAN'T RECALL

[Q2] Did you ever read this message?

*If Respondent did not read the text messages, let him read the text message and continue with questions*

YES NO

NOT SURE CAN'T RECALL

YES NO

NOT SURE CAN'T RECALL

[Q4] Could you please indicate why this text message is diﬃcult or not easy to understand/ unclear?

*NA--999*

[Q5] In your own few words can you tell me how you understood this message?

*NA--999*

[Q6] Was this SMS text too long/wordy?

YES NO

NOT SURE SOMEHOW DON'T KNOW

[Q7] Would you like to reword/rewrite the text message?

YES NO

NOT APPLICABLE

[Q8] How would you want to reword this message?

*(provide a sticker for rewording or write in the space below) NA---999*

[Q9] Do you think that this SMS text would be relevant in increasing uptake of Breast cancer screening?

YES NO

Message CC16

"Your life can be saved if Cervical cancer is detected early. Go for screening"

*( FW should read the text messages or Show Message Log to Respondent)*

[Q1] Did you ever receive this message?

YES NO

NOT SURE CAN'T RECALL

[Q2] Did you ever read this message?

*If Respondent did not read the text messages, let him read the text message and continue with questions*

YES NO

NOT SURE CAN'T RECALL

[Q3] Was the text message easy to understand/clear?

YES NO

NOT SURE CAN'T RECALL

[Q4] Could you please indicate why this text message is diﬃcult or not easy to understand/ unclear?

*NA--999*

[Q5] In your own few words can you tell me how you understood this message?

*NA--999*

[Q6] Was this SMS text too long/wordy?

YES NO

NOT SURE SOMEHOW DON'T KNOW

[Q7] Would you like to reword/rewrite the text message?

YES NO

NOT APPLICABLE

[Q8] How would you want to reword this message?

*(provide a sticker for rewording or write in the space below) NA---999*

[Q9] Do you think that this SMS text would be relevant in increasing uptake of Breast cancer screening?

YES NO

Message CC17

"Do not let Cervical cancer kill your dreams. Go for screening!"

*( FW should read the text messages or Show Message Log to Respondent)*

[Q1] Did you ever receive this message?

YES NO

NOT SURE CAN'T RECALL

[Q2] Did you ever read this message?

*If Respondent did not read the text messages, let him read the text message and continue with questions*

YES NO

NOT SURE CAN'T RECALL

[Q3] Was the text message easy to understand/clear?

YES NO

NOT SURE CAN'T RECALL

[Q4] Could you please indicate why this text message is diﬃcult or not easy to understand/ unclear?

*NA--999*

[Q5] In your own few words can you tell me how you understood this message?

*NA--999*

[Q6] Was this SMS text too long/wordy?

YES NO

NOT SURE SOMEHOW DON'T KNOW

[Q7] Would you like to reword/rewrite the text message?

YES NO

NOT APPLICABLE

[Q8] How would you want to reword this message?

*(provide a sticker for rewording or write in the space below) NA---999*

[Q9] Do you think that this SMS text would be relevant in increasing uptake of Breast cancer screening?

YES NO

Message CC18

"Ghana needs you alive. Go for cervical cancer screening today!"

*( FW should read the text messages or Show Message Log to Respondent)*

[Q1] Did you ever receive this message?

YES NO

NOT SURE CAN'T RECALL

[Q2] Did you ever read this message?

*If Respondent did not read the text messages, let him read the text message and continue with questions*

YES NO

NOT SURE CAN'T RECALL

YES NO

NOT SURE CAN'T RECALL

[Q4] Could you please indicate why this text message is diﬃcult or not easy to understand/ unclear?

*NA--999*

[Q5] In your own few words can you tell me how you understood this message?

*NA--999*

[Q6] Was this SMS text too long/wordy?

YES NO

NOT SURE SOMEHOW DON'T KNOW

[Q7] Would you like to reword/rewrite the text message?

YES NO

NOT APPLICABLE

[Q8] How would you want to reword this message?

*(provide a sticker for rewording or write in the space below) NA---999*

[Q9] Do you think that this SMS text would be relevant in increasing uptake of Breast cancer screening?

YES NO

Message CC19

"Early detection of Cervical cancer reduces cost of treatment. Go for screening!"

*( FW should read the text messages or Show Message Log to Respondent)*

[Q1] Did you ever receive this message?

YES NO

NOT SURE CAN'T RECALL

[Q2] Did you ever read this message?

*If Respondent did not read the text messages, let him read the text message and continue with questions*

YES NO

NOT SURE CAN'T RECALL

[Q3] Was the text message easy to understand/clear?

YES NO

NOT SURE CAN'T RECALL

[Q4] Could you please indicate why this text message is diﬃcult or not easy to understand/ unclear?

*NA--999*

[Q5] In your own few words can you tell me how you understood this message?

*NA--999*

[Q6] Was this SMS text too long/wordy?

YES NO

NOT SURE SOMEHOW DON'T KNOW

[Q7] Would you like to reword/rewrite the text message?

YES NO

NOT APPLICABLE

[Q8] How would you want to reword this message?

*(provide a sticker for rewording or write in the space below) NA---999*

[Q9] Do you think that this SMS text would be relevant in increasing uptake of Breast cancer screening?

YES NO

Message CC20

"Cervical cancer screening is not harmful! Go for cervical cancer screening today!"

*( FW should read the text messages or Show Message Log to Respondent)*

[Q1] Did you ever receive this message?

YES NO

NOT SURE CAN'T RECALL

[Q2] Did you ever read this message?

*If Respondent did not read the text messages, let him read the text message and continue with questions*

YES NO

NOT SURE CAN'T RECALL

[Q3] Was the text message easy to understand/clear?

YES NO

NOT SURE CAN'T RECALL

[Q4] Could you please indicate why this text message is diﬃcult or not easy to understand/ unclear?

*NA--999*

[Q5] In your own few words can you tell me how you understood this message?

*NA--999*

[Q6] Was this SMS text too long/wordy?

YES NO

NOT SURE SOMEHOW DON'T KNOW

[Q7] Would you like to reword/rewrite the text message?

YES NO

NOT APPLICABLE

[Q8] How would you want to reword this message?

*(provide a sticker for rewording or write in the space below) NA---999*

[Q9] Do you think that this SMS text would be relevant in increasing uptake of Breast cancer screening?

YES NO

Message CC21

"Cervical cancer is curable when detected early. Go for Cervical cancer screening today!"

*( FW should read the text messages or Show Message Log to Respondent)*

[Q1] Did you ever receive this message?

YES NO

NOT SURE CAN'T RECALL

[Q2] Did you ever read this message?

*If Respondent did not read the text messages, let him read the text message and continue with questions*

YES NO

NOT SURE CAN'T RECALL

YES NO

NOT SURE CAN'T RECALL

[Q4] Could you please indicate why this text message is diﬃcult or not easy to understand/ unclear?

*NA--999*

[Q5] In your own few words can you tell me how you understood this message?

*NA--999*

[Q6] Was this SMS text too long/wordy?

YES NO

NOT SURE SOMEHOW DON'T KNOW

[Q7] Would you like to reword/rewrite the text message?

YES NO

NOT APPLICABLE

[Q8] How would you want to reword this message?

*(provide a sticker for rewording or write in the space below) NA---999*

[Q9] Do you think that this SMS text would be relevant in increasing uptake of Breast cancer screening?

YES NO

Message CC22

"8 out of every 10 women who have their cervix checked regularly are saved from cervical cancer and prevented from cervical cancer death. Go for screening!"

*( FW should read the text messages or Show Message Log to Respondent)*

[Q1] Did you ever receive this message?

YES NO

NOT SURE CAN'T RECALL

[Q2] Did you ever read this message?

*If Respondent did not read the text messages, let him read the text message and continue with questions*

YES NO

NOT SURE CAN'T RECALL

[Q3] Was the text message easy to understand/clear?

YES NO

NOT SURE CAN'T RECALL

[Q4] Could you please indicate why this text message is diﬃcult or not easy to understand/ unclear?

*NA--999*

[Q5] In your own few words can you tell me how you understood this message?

*NA--999*

[Q6] Was this SMS text too long/wordy?

YES NO

NOT SURE SOMEHOW DON'T KNOW

[Q7] Would you like to reword/rewrite the text message?

YES NO

NOT APPLICABLE

[Q8] How would you want to reword this message?

*(provide a sticker for rewording or write in the space below) NA---999*

[Q9] Do you think that this SMS text would be relevant in increasing uptake of Breast cancer screening?

YES NO

Message CC23

"9 out of every 10 women who have early detection and treatment for cervical cancer are still alive after 5 years. Go for screening!"

*( FW should read the text messages or Show Message Log to Respondent)*

[Q1] Did you ever receive this message?

YES NO

NOT SURE CAN'T RECALL

[Q2] Did you ever read this message?

*If Respondent did not read the text messages, let him read the text message and continue with questions*

YES NO

NOT SURE CAN'T RECALL

[Q3] Was the text message easy to understand/clear?

YES NO

NOT SURE CAN'T RECALL

[Q4] Could you please indicate why this text message is diﬃcult or not easy to understand/ unclear?

*NA--999*

[Q5] In your own few words can you tell me how you understood this message?

*NA--999*

[Q6] Was this SMS text too long/wordy?

YES NO

NOT SURE SOMEHOW DON'T KNOW

[Q7] Would you like to reword/rewrite the text message?

YES NO

NOT APPLICABLE

[Q8] How would you want to reword this message?

*(provide a sticker for rewording or write in the space below) NA---999*

[Q9] Do you think that this SMS text would be relevant in increasing uptake of Breast cancer screening?

YES NO

Message CC24

"Having Cervical cancer is not a death sentence. It can be treated. Go for screening!"

*( FW should read the text messages or Show Message Log to Respondent)*

[Q1] Did you ever receive this message?

YES NO

NOT SURE CAN'T RECALL

[Q2] Did you ever read this message?

*If Respondent did not read the text messages, let him read the text message and continue with questions*

YES NO

NOT SURE CAN'T RECALL

YES NO

NOT SURE CAN'T RECALL

[Q4] Could you please indicate why this text message is diﬃcult or not easy to understand/ unclear?

*NA--999*

[Q5] In your own few words can you tell me how you understood this message?

*NA--999*

[Q6] Was this SMS text too long/wordy?

YES NO

NOT SURE SOMEHOW DON'T KNOW

[Q7] Would you like to reword/rewrite the text message?

YES NO

NOT APPLICABLE

[Q8] How would you want to reword this message?

*(provide a sticker for rewording or write in the space below) NA---999*

[Q9] Do you think that this SMS text would be relevant in increasing uptake of Breast cancer screening?

YES NO

Message CC25

"Your Health is your Wealth. Make time for your Health. Go for cervical cancer screening!"

*( FW should read the text messages or Show Message Log to Respondent)*

[Q1] Did you ever receive this message?

YES NO

NOT SURE CAN'T RECALL

[Q2] Did you ever read this message?

*If Respondent did not read the text messages, let him read the text message and continue with questions*

YES NO

NOT SURE CAN'T RECALL

[Q3] Was the text message easy to understand/clear?

YES NO

NOT SURE CAN'T RECALL

[Q4] Could you please indicate why this text message is diﬃcult or not easy to understand/ unclear?

*NA--999*

[Q5] In your own few words can you tell me how you understood this message?

*NA--999*

[Q6] Was this SMS text too long/wordy?

YES NO

NOT SURE SOMEHOW DON'T KNOW

[Q7] Would you like to reword/rewrite the text message?

YES NO

NOT APPLICABLE

[Q8] How would you want to reword this message?

*(provide a sticker for rewording or write in the space below) NA---999*

[Q9] Do you think that this SMS text would be relevant in increasing uptake of Breast cancer screening?

YES NO

Message CC26

"Have time for cervical cancer screening today. Save your life tomorrow. Spend time on your health. It is not time wasted."

*( FW should read the text messages or Show Message Log to Respondent)*

[Q1] Did you ever receive this message?

YES NO

NOT SURE CAN'T RECALL

[Q2] Did you ever read this message?

*If Respondent did not read the text messages, let him read the text message and continue with questions*

YES NO

NOT SURE CAN'T RECALL

[Q3] Was the text message easy to understand/clear?

YES NO

NOT SURE CAN'T RECALL

[Q4] Could you please indicate why this text message is diﬃcult or not easy to understand/ unclear?

*NA--999*

[Q5] In your own few words can you tell me how you understood this message?

*NA--999*

[Q6] Was this SMS text too long/wordy?

YES NO

NOT SURE SOMEHOW DON'T KNOW

[Q7] Would you like to reword/rewrite the text message?

YES NO

NOT APPLICABLE

[Q8] How would you want to reword this message?

*(provide a sticker for rewording or write in the space below) NA---999*

[Q9] Do you think that this SMS text would be relevant in increasing uptake of Breast cancer screening?

YES NO

Message CC27

"Take time oﬀ your busy schedules and go for cervical cancer screening. A short time spent on cervical cancer screening saves a long stay at the hospital for treatment."

*( FW should read the text messages or Show Message Log to Respondent)*

[Q1] Did you ever receive this message?

YES NO

NOT SURE CAN'T RECALL

[Q2] Did you ever read this message?

*If Respondent did not read the text messages, let him read the text message and continue with questions*

YES NO

NOT SURE CAN'T RECALL

YES NO

NOT SURE CAN'T RECALL

[Q4] Could you please indicate why this text message is diﬃcult or not easy to understand/ unclear?

*NA--999*

[Q5] In your own few words can you tell me how you understood this message?

*NA--999*

[Q6] Was this SMS text too long/wordy?

YES NO

NOT SURE SOMEHOW DON'T KNOW

[Q7] Would you like to reword/rewrite the text message?

YES NO

NOT APPLICABLE

[Q8] How would you want to reword this message?

*(provide a sticker for rewording or write in the space below) NA---999*

[Q9] Do you think that this SMS text would be relevant in increasing uptake of Breast cancer screening?

YES NO

Message CC28

"Have you had your cervical cancer screening? Make time for it today!"

*( FW should read the text messages or Show Message Log to Respondent)*

[Q1] Did you ever receive this message?

YES NO

NOT SURE CAN'T RECALL

[Q2] Did you ever read this message?

*If Respondent did not read the text messages, let him read the text message and continue with questions*

YES NO

NOT SURE CAN'T RECALL

[Q3] Was the text message easy to understand/clear?

YES NO

NOT SURE CAN'T RECALL

[Q4] Could you please indicate why this text message is diﬃcult or not easy to understand/ unclear?

*NA--999*

[Q5] In your own few words can you tell me how you understood this message?

*NA--999*

[Q6] Was this SMS text too long/wordy?

YES NO

NOT SURE SOMEHOW DON'T KNOW

[Q7] Would you like to reword/rewrite the text message?

YES NO

NOT APPLICABLE

[Q8] How would you want to reword this message?

*(provide a sticker for rewording or write in the space below) NA---999*

[Q9] Do you think that this SMS text would be relevant in increasing uptake of Breast cancer screening?

YES NO

Message CC29

"Cervical cancer screening does not take long. You spend less time testing for Cervical cancer than you spend at the hairdresser's"

*( FW should read the text messages or Show Message Log to Respondent)*

[Q1] Did you ever receive this message?

YES NO

NOT SURE CAN'T RECALL

[Q2] Did you ever read this message?

*If Respondent did not read the text messages, let him read the text message and continue with questions*

YES NO

NOT SURE CAN'T RECALL

[Q3] Was the text message easy to understand/clear?

YES NO

NOT SURE CAN'T RECALL

[Q4] Could you please indicate why this text message is diﬃcult or not easy to understand/ unclear?

*NA--999*

[Q5] In your own few words can you tell me how you understood this message?

*NA--999*

[Q6] Was this SMS text too long/wordy?

YES NO

NOT SURE SOMEHOW DON'T KNOW

[Q7] Would you like to reword/rewrite the text message?

YES NO

NOT APPLICABLE

[Q8] How would you want to reword this message?

*(provide a sticker for rewording or write in the space below) NA---999*

[Q9] Do you think that this SMS text would be relevant in increasing uptake of Breast cancer screening?

YES NO

Message CC30

"Cervical cancer screening is available in Ghana. You can visit any of the following facilities;

1. Tema General Hospital, Tema
2. Narh-Bita Hospital, Tema
3. Sinel Specialist Hospital, Tema
4. Korle-Bu Teaching Hospital, Korle-Bu
5. Ridge Hospital, Accra"

*( FW should read the text messages or Show Message Log to Respondent)*

[Q1] Did you ever receive this message?

YES NO

NOT SURE CAN'T RECALL

[Q2] Did you ever read this message?

*If Respondent did not read the text messages, let him read the text message and continue with questions*

YES NO

NOT SURE CAN'T RECALL

NO

NOT SURE CAN'T RECALL

[Q4] Could you please indicate why this text message is diﬃcult or not easy to understand/ unclear?

*NA--999*

[Q5] In your own few words can you tell me how you understood this message?

*NA--999*

[Q6] Was this SMS text too long/wordy?

YES NO

NOT SURE SOMEHOW DON'T KNOW

[Q7] Would you like to reword/rewrite the text message?

YES NO

NOT APPLICABLE

[Q8] How would you want to reword this message?

*(provide a sticker for rewording or write in the space below) NA---999*

[Q9] Do you think that this SMS text would be relevant in increasing uptake of Breast cancer screening?

YES NO

Message CC31

"The cost of Cervical cancer screening is far less than the cost for treatment. Go for screening today!"

*( FW should read the text messages or Show Message Log to Respondent)*

NO

NOT SURE CAN'T RECALL

[Q2] Did you ever read this message?

*If Respondent did not read the text messages, let him read the text message and continue with questions*

YES NO

NOT SURE CAN'T RECALL

[Q3] Was the text message easy to understand/clear?

YES NO

NOT SURE CAN'T RECALL

[Q4] Could you please indicate why this text message is diﬃcult or not easy to understand/ unclear?

*NA--999*

[Q5] In your own few words can you tell me how you understood this message?

*NA--999*

[Q6] Was this SMS text too long/wordy?

YES NO

NOT SURE SOMEHOW DON'T KNOW

[Q7] Would you like to reword/rewrite the text message?

YES NO

NOT APPLICABLE

[Q8] How would you want to reword this message?

*(provide a sticker for rewording or write in the space below) NA---999*

[Q9] Do you think that this SMS text would be relevant in increasing uptake of Breast cancer screening?

YES NO

Message CC32

"It is better to spend your pocket money today on cervical cancer screening than to spend your life savings on treatment tomorrow. Go for screening today!"

*( FW should read the text messages or Show Message Log to Respondent)*

[Q1] Did you ever receive this message?

YES NO

NOT SURE CAN'T RECALL

[Q2] Did you ever read this message?

*If Respondent did not read the text messages, let him read the text message and continue with questions*

YES NO

NOT SURE CAN'T RECALL

[Q3] Was the text message easy to understand/clear?

YES NO

NOT SURE CAN'T RECALL

[Q4] Could you please indicate why this text message is diﬃcult or not easy to understand/ unclear?

*NA--999*

[Q5] In your own few words can you tell me how you understood this message?

*NA--999*

[Q6] Was this SMS text too long/wordy?

YES NO

NOT SURE SOMEHOW DON'T KNOW

[Q7] Would you like to reword/rewrite the text message?

YES NO

NOT APPLICABLE

[Q8] How would you want to reword this message?

*(provide a sticker for rewording or write in the space below) NA---999*

[Q9] Do you think that this SMS text would be relevant in increasing uptake of Breast cancer screening?

YES NO

SECTION B- GENERAL QUESTIONS

I would like to ask you some general questions about ALL the text messages.

[B10] How often do you read SMS texts messages on your phone?

Before close of day By mid-week (2-3 days)

By close of week (4-7 days) Not at all

Other

[B10_other] Other specify

[B11] One the average, can you please indicate how long it took you to read the text messages you received from UG-SPH?

Immediately after receipt (less than 1 hour) Within 1 –4 hours after receipt of message Within 4-8 hours after receipt of message At the end of each day of receipt

Within 2-3 Days of receipt

Within a week of receipt of message Did not read any

Other

[B11_other] Other specify

[b12] On the average, did you read most of the text messages during working hours, break times or after close of work?

YES NO

[b12_1] During working hours [b12_2] After close of work [b12_3] During break, snack or lunch time [b12_4] Other

[B12_other] Other specify

[b13] What was your experience being on this programme?

[b14.1] What is most engaging about this programme?

[b14.2] What is least engaging about this programme?

[b15] Is there any confusing/irritating aspect of this programme?

YES NO

CAN'T TELL

[b16] Generally, was the text message volume adequate?

YES NO

[b17] Generally was the timing of messages appropriate?

YES NO

CAN'T TELL

[b18] If no, would you like to suggest a timing schedule?

YES NO

» [b19] If Yes, kindly provide a timing schedule

Time in Morning

hh:mm

Time in Evening

hh:mm

[b20] Did the SMS text messages inﬂuence your views on Breast cancer screening?

YES NO

[b21] Do you have challenges with the mobile network coverage during working hours?

YES NO

[b22] Do you have access to your phone during working hours?

YES NO

[b23] Is it acceptable or not acceptable to you for information on Breast cancer screening to be provided via SMS text messages?*

YES NO

[b24] Would you like the National Non-Communicable Diseases Control Program to send SMS text reminders on Breast cancer screening on your personal phones in the future?

YES NO

Time interview Ended

hh:mm

WE HAVE NOW COME TO THE END OF THIS SURVEY. THANK YOU VERY MUCH FOR PARTICIPATING!

INTWER: ENTER FIELD NOTES/COMMENTS

INTWER: TAKE GPS

latitude (x.y °)

|  | | | |
| --- | --- | --- | --- |
|  | Map | [Report a map error](https://www.google.com/maps/%405.6081841%2C-0.2058183%2C15z/data%3D!10m1!1e1!12b1?source=apiv3&rapsrc=apiv3) |  |

longitude (x.y °)

altitude (m)

accuracy (m)

data [©2018 Google](https://www.google.com/maps/%405.6081841%2C-0.2058183%2C15z/data%3D!10m1!1e1!12b1?source=apiv3&rapsrc=apiv3)
