# Supplementary material for: Developing a culturally tailored short message service (SMS) intervention for improving the uptake of cervical cancer screening among Ghanaian women in urban communities
Source: BMC Womens Health. 2022 May 10;22:154. doi: 10.1186/s12905-022-01719-9 (PMC9092690; doi:10.1186/s12905-022-01719-9)
Supplement: Supplementary file 5 — Additional file 5. Type of information to be included in SMS messages. [file 12905_2022_1719_MOESM5_ESM.docx]

“**Supplementary file s5: Type of information on CC screening to be included in text messages**

| **Characteristic** | **Freq.** | **Percent** |
| --- | --- | --- |
| What cervical cancer is | 7 | 5.4 |
| What cervical cancer is, Causes/Risk factors | 2 | 1.5 |
| What cervical cancer is, Causes/Risk factors, Prevention | 10 | 7.7 |
| What cervical cancer is, Causes/Risk factors, Prevention, Treatment | 10 | 7.7 |
| What cervical cancer is, Causes/Risk factors, Prevention, Treatment, Importance of CC | 6 | 4.6 |
| What cervical cancer is, Causes/Risk factors, Prevention, Treatment, Importance of CC, Cost of CC test | 1 | 0.8 |
| What cervical cancer is, Causes/Risk factors, Prevention, Treatment, Importance of CC, What to do if CC test is positive | 1 | 0.8 |
| What cervical cancer is, Causes/Risk factors, Prevention, Treatment, Importance of CC, other | 1 | 0.8 |
| What cervical cancer is, Causes/Risk factors, Prevention, Treatment, Where to go for CC testing | 1 | 0.8 |
| What cervical cancer is, Causes/Risk factors, Prevention, Importance of CC | 3 | 2.3 |
| What cervical cancer is, Causes/Risk factors, Prevention, Importance of CC, Other | 4 | 3.1 |
| What cervical cancer is, Causes/Risk factors, Prevention, Where to go for CC test, Cost of CC test | 1 | 0.8 |
| What cervical cancer is, Causes/Risk factors, Prevention, Cost of CC test | 1 | 0.8 |
| What cervical cancer is, Causes/Risk factors, Prevention, What to do if CC test is positive | 1 | 0.8 |
| What cervical cancer is, Causes/Risk factors, Prevention, other | 8 | 6.2 |
| What cervical cancer is, Causes/Risk factors, Treatment | 1 | 0.8 |
| What cervical cancer is, Causes/Risk factors, Treatment, Importance of CC test | 1 | 0.8 |
| Importance of CC testing, other | 1 | 0.8 |
| What cervical cancer is, Causes/Risk factors, Treatment, Cost of CC testing | 1 | 0.8 |
| What cervical cancer is, Causes/Risk factors, Where to go for CC tests | 1 | 0.8 |
| What cervical cancer is, Causes/Risk factors, What to do if Mammogram is positive, Other | 1 | 0.8 |
| What cervical cancer is, Causes/Risk factors, Other | 3 | 2.3 |
| What cervical cancer is, Prevention | 1 | 0.8 |
| What cervical cancer is, Prevention, Treatment, Importance of CC testing | 1 | 0.8 |
| What cervical cancer is, Prevention, Treatment, Importance of CC testing, Other | 1 | 0.8 |
| What cervical cancer is, Prevention, Treatment, Cost of CC testing | 1 | 0.8 |
| What cervical cancer is, Prevention, Cost of CC testing | 2 | 1.5 |
| What cervical cancer is, Prevention, Other | 1 | 0.8 |
| What cervical cancer is, Treatment | 1 | 0.8 |
| What cervical cancer is, Treatment, Where to go for CC testing | 1 | 0.8 |
| What cervical cancer is, Other | 4 | 3.1 |
| Causes/Risk factors | 5 | 3.8 |
| Causes/Risk factors, Prevention | 9 | 6.9 |
| Causes/Risk factors, Prevention, Treatment | 7 | 5.4 |
| Causes/Risk factors, Prevention, Treatment, Where to go for CC testing | 1 | 0.8 |
| Causes/Risk factors, Prevention, Importance of CC testing | 1 | 0.8 |
| Causes/Risk factors, Prevention, Importance of CC testing, Where to go for CC testing | 1 | 0.8 |
| Causes/Risk factors, Prevention, Importance of CC testing, Where to go for CC testing, Cost of CC tests | 1 | 0.8 |
| Causes/Risk factors, Prevention, Where to go for CC tests, Cost of CC test, Other | 1 | 0.8 |
| Causes/Risk factors, Prevention, other | 2 | 1.5 |
| Causes/Risk factors, Treatment | 2 | 1.5 |
| Causes/Risk factors, Treatment, Importance of CC testing, What to do if CC test is positive | 1 | 0.8 |
| Causes/Risk factors, Treatment, other | 2 | 1.5 |
| Causes/Risk factors, Importance of CC tests | 1 | 0.8 |
| Prevention, Cost of CC tests, other | 1 | 0.8 |
| Importance of CC tests | 1 | 0.8 |
| Other | 15 | 11.5 |
| **Total** | **130** | **100.0** |
